# Characterization of Microbial Diversity in Decayed Wood from a Spanish Forest: An Environmental Source of Industrially Relevant Microorganisms

**DOI:** 10.3390/microorganisms10061249

**Published:** 2022-06-18

**Authors:** Óscar Velasco-Rodríguez, Mariana Fil, Tonje M. B. Heggeset, Kristin F. Degnes, David Becerro-Recio, Katarina Kolsaková, Tone Haugen, Malene Jønsson, Macarena Toral-Martínez, Carlos García-Estrada, Alberto Sola-Landa, Kjell D. Josefsen, Håvard Sletta, Carlos Barreiro

**Affiliations:** 1 INBIOTEC (Instituto de Biotecnología de León), Avda Real 1, 24006 León, Spain; ovelr@unileon.es (Ó.V.-R.); mariana.fil@inbiotec.com (M.F.); davidbecerro92@gmail.com (D.B.-R.); katarina.kosalkova@inbiotec.com (K.K.); mtoralm00@gmail.com (M.T.-M.); c.gestrada@unileon.es (C.G.-E.); alberto.sola@inbiotec.com (A.S.-L.); 2 SINTEF Industry, Department of Biotechnology and Nanomedicine, P.O. Box 4760 Torgarden, N-7465 Trondheim, Norway; tonje.heggeset@sintef.no (T.M.B.H.); kristin.f.degnes@sintef.no (K.F.D.); tone.haugen@sintef.no (T.H.); malene.l.jonsson@gmail.com (M.J.); kjell.d.josefsen@sintef.no (K.D.J.); havard.sletta@sintef.no (H.S.); 3 Departamento de Ciencias Biomédicas, Universidad de León, Campus de Vegazana, s/n, 24007 León, Spain; 4 Área de Bioquímica y Biología Molecular, Departamento de Biología Molecular, Universidad de León, Campus de Vegazana, 24071 León, Spain

**Keywords:** fungi, bacteria, wood decay, rotten wood, secondary metabolites, antibiotic, polyketide synthase (PKS), non-ribosomal peptide synthetase (NRPS), cellulase, esterase, ferulic acid, enzyme

## Abstract

Rotting wood is inhabited by a large diversity of bacteria, fungi, and insects with complex environmental relationships. The aim of this work was to study the composition of the microbiota (bacteria and fungi) in decaying wood from a northwest Spanish forest as a source of industrially relevant microorganisms. The analyzed forest is situated in a well-defined biogeographic area combining Mediterranean and temperate macrobioclimates. Bacterial diversity, determined by metagenome analyses, was higher than fungal heterogeneity. However, a total of 194 different cultivable bacterial isolates (mainly *Bacillaceae*, *Streptomycetaceae*, *Paenibacillaceae*, and *Microbacteriaceae*) were obtained, in contrast to 343 fungal strains (mainly *Aspergillaceae*, *Hypocreaceae*, and *Coniochaetaceae*). Isolates traditionally known as secondary metabolite producers, such as Actinobacteria and members of the *Penicillium* genus, were screened for their antimicrobial activity by the detection of antibiotic biosynthetic clusters and competitive bioassays against fungi involved in wood decay. In addition, the ability of *Penicillium* isolates to degrade cellulose and release ferulic acid from wood was also examined. These results present decaying wood as an ecologically rich niche and a promising source of biotechnologically interesting microorganisms.

## 1. Introduction

Wood consists mainly of cellulose (40–60%), hemicelluloses (15–30%), and lignin (17–35%), depending on wood species [1], and is a rich source of carbon in nature. However, wood is not an easily biodegradable substance, due to its low water content, as well as its hard-to-degrade lignin and the crystalline cellulose structures. The content of other nutrients, such as phosphorus, potassium, and especially nitrogen, is low.

Deadwood represents a specific habitat in the forest and can exceed the biomass of living trees [2]. It provides shelter and nutrition to a great variety of organisms, including fungi, bacteria, and saprophytic insects, and subsequently, its degradation generates nutrients for new plant growth and energy to animals [3,4]. The fungal processes and diversity, as main wood degraders, have been broadly studied over the years, but the presence and role of prokaryotes in deadwood degradation is still poorly understood [5,6]. Based on their mode of action during wood breakdown, fungi can be classified as: (i) soft rot fungi, which degrade cellulose and hemicelluloses, but cannot degrade lignin, and cause a decrease in impact bending strength and a reduction in dimensional stability [4], (ii) white rot fungi, which are able to degrade cellulose, hemicelluloses, and lignin simultaneously or successively [7], and (iii) brown rot fungi that degrade cellulose and hemicelluloses of the wood cell walls and modify lignin [8]. Degradation can also modify the appearance of wood bricks and change their physical and mechanical properties. In addition to the degradation of the vegetal cell components, the discoloration of wood by fungi, bacteria, or algae is also a long-known problem (e.g., blue stain) [9].

Basidiomycete fungi are the main initial microbial decomposers of lignocellulosic biomass, and they can degrade or modify all the components of wood by enzymatic and non-enzymatic processes [5]. As soon as the initial degradation processes have broken up the wood structure, the lignin protection of cellulose and hemicelluloses against enzymatic degradation is reduced, and water can penetrate deeper into the wood. This enables other microorganisms, different from the initial wood degraders, to gain a foothold [10]. These complex biological interactions create a highly interesting environmental niche in which to look for microorganism with potential industrial applications in red (e.g., antibiotics, drugs) and white (e.g., enzymes) biotechnologies. Although this niche has not been deeply investigated as a source of antimicrobial compounds, some wood-decaying fungi, or fungi associated with them, present antimicrobial activities [11,12,13]. Besides, different bioactive potential has been described in microorganisms isolated from decaying wood, such as the antimicrobial biosynthetic capacity in *Paraburkholderia* sp., or anti-insect activity from alkaloids isolated from *Penicillium* species [14,15]. This suggest rotten wood as a relevant niche to find more and novel antimicrobial producing microorganisms.

Antibiotics are antimicrobial agents, commonly used to treat infection, but which are also used as biological weapons by their producers (certain bacteria and fungi) to subsist in multispecies environments where nutrients may be limited. Thus, researchers have focused on exploring new, unusual, or extreme environments, as well as developing faster screening methods, to identify undiscovered microorganisms encoding novel biosynthetic gene clusters for bioactive molecules [16]. Many publications from recent decades report studies of the diversity and biosynthetic potential of microorganisms from sources such as sea sponges, corals, medical plants from tropical rain forests, geothermal springs, desert rocks, and microorganism associated with lichen symbiosis [17,18,19,20,21,22,23]. This fuels the screening of new environmental niches as putative sources of novel bioactive molecules, as demonstrated by the discovery of a new molecule, cyphomycin, produced by a *Streptomyces* species isolated from the microbiome of ants, which showed a high activity against multidrug resistant fungal pathogens [24].

Enzymes are among the beneficial compounds produced by microorganisms with widespread use in white biotechnology. Cellulases hydrolyse cellulose (a globally abundant renewable biopolymer) into fermentable sugars that can be used to produce commercially relevant products, such as ethanol, organic acids, and animal feed [9]. The principal microbial producers of this class of enzymes are fungi, mainly *Aspergillus*, *Rhizopus*, *Trichoderma*, *Fusarium*, *Neurospora*, and *Penicillium*, but also bacteria such as *Clostridium*, *Cellulomonas*, *Bacillus*, and *Pseudomonas* [25,26]. Although the global enzyme market has been dominated by enzymes isolated from the *Aspergillus* and *Trichoderma* species, properties such as the lack of glucosidase activity in *Trichoderma reesei*, one of the main species used on the production of cellulases, have changed the focus to other genera exhibiting lignocellulosic biomass-degrading enzymes, such as *Penicillium* [27,28].

Hydrolysis of plant biomass can release useful compounds, such as hydroxycinnamic acids. One way to do this it is by using feruloyl esterase enzymes, which are auxiliary enzymes that hydrolyse the ester bonds that join the hydroxycinnamic acids to the lignin-carbohydrate complex, enabling extraction of this useful compound [29,30]. Among the plant hydrolysate products, ferulic acid has industrial and medical applications. The antioxidative activity of ferulic acid can be used in medicine to reduce the oxidative stress caused by reactive oxygen species. Some studies have proposed the used of ferulic acid in cancer treatment, and also as anti-inflammatory, antimicrobial, antiviral, etc. [31]. This organic acid is also being used in other fields, such as the cosmetic industry, where, due to its antioxidative properties, it is used in anti-aging or solar protective formulations, and in the food industry, where it is a precursor of the well know flavouring compound vanillin [32].

This study evaluated the microbiota of rotting wood through metabarcode sequencing, while the cultivable microorganisms were characterized by traditional microbiological methods. Finally, the industrial biosynthetic potential of the isolated microorganisms for drug and enzyme production, and their activity as biocontrol agents, were validated.

## 2. Materials and Methods

### 2.1. Sampling of Decayed Wood

Fourteen individual wood samples were collected from the forest of Villavieja (León, Spain: 42.485015, −6.684764, 765 MASL) in June 2017, (Supplementary Table S1). The village is in northwest Spain inside a clear biogeographical region (*Hoya del Bierzo*), recognized by the worldwide bioclimatic classification system, because it is a well delimited territory in geographical terms containing series of species, associations, and particularly, specific topographical geo segments [33].

The tree genus/species was determined by visual inspection, and the degree of decay was evaluated by appearance and texture using the Swedish National Forest Inventory (NFI) classification system, with scores between 1 (low degree of decay) and 4 (high degree of decay) [34].

Each sample from each tree was harvested using a sterile saw and brought back to the lab in one piece on cooling elements. Samples for isolation of cultivable microbes were processed as described below, whereas samples for metabarcode sequencing were packed in zippered plastic bags and shipped to SINTEF labs (Trondheim, Norway) on cooling elements. Immediately after arrival in Norway, the wood pieces were first cut into smaller pieces using a drill, tweezers, a knife, and/or a hammer and then homogenized using a coffee grinder. All the equipment was sterilized between each sample. The coffee grinder was washed with a wet paper, and then sterilized with 100% ethanol that was evaporated in a sterile hood. The other equipment was washed with water, dried, and then sterilized using 70% ethanol that was burned off by a flame. To obtain a fine powder for DNA extraction, grinding with liquid nitrogen in a sterile mortar was performed.

### 2.2. DNA Extraction for Metagenome Analyses

DNA was isolated by Cetyl Trimethyl Ammonium Bromide (CTAB)-mediated chloroform-isoamyl alcohol extraction. In brief: 0.1 g of homogenized wood sample was transferred to a 2 mL bead-beater tube containing glass beads (∅ 0.5 mm) and 0.5 mL DNA extraction buffer (100 mM Tris-HCl, 100 mM sodium EDTA, 100 mM sodium phosphate (pH 8.0), 1.5 M NaCl, 1% CTAB). Samples were incubated on a VortexGenie 2.0 at maximum speed (5 min, room temperature) followed by bead-beating (1 min, 30 Hz) in a Tissuelyser II (Qiagen, Hilden, Germany). A total of 10 µL of Proteinase K (10 mg/mL) were added and the mixtures, which were incubated in a horizontal shaker (125 rpm, 37 °C, 30 min). Then, 0.05 mL of SDS (20%) were added, and the mixtures were incubated at 65 °C (2 h, gently mixed at least every 15 min). After cell lysis, solids were precipitated by centrifugation, and DNA was extracted twice with equal volumes of chloroform:isoamylalcohol (24:1). The upper phase containing the DNA was then precipitated with 0.6 volumes of cold isopropanol. DNA was recovered by centrifugation, washed with ice-cold ethanol (70%), and finally dissolved in 100 µL elution buffer (10 mM Tris-Cl, pH 8.5). DNA quality was evaluated by microvolume measurements on a DeNovix DS-11 fx+, and DNA concentration was determined by the Qubit^TM^ dsDNA BR Assay kit (Life technologies AS, Lillestrøm, Norway).

### 2.3. Metagenome Sequencing

Sequencing amplicon libraries were generated by PCR following the 16S Metagenomic Sequencing Library Preparation, Preparing 16S Ribosomal RNA Gene Amplicons for the Illumina MiSeq System protocol (Illumina part number 15044223 rev. B) with primers targeting bacteria or fungi, respectively (Table 1). In brief, internal parts of the 16S ribosomal RNA (rRNA) gene, covering variable regions V3 and V4, of bacteria, or the internal transcribed spacer (ITS2) of fungi were PCR-amplified with the KAPA HiFi HotStart ReadyMix (KAPA Biosystems, Wilmington, MA, USA). PCR products were purified with the Agencourt AMPure XP kit (Beckman Coulter Genomics, Newton, MA, USA). The Nextera XT Index Kit was used to add sequencing adapters and multiplexing indices. Pooled DNA libraries were sequenced on a MiSeq sequencer (Illumina, Eindhoven, Netherlands) using the MiSeq Reagent Kit v3 in the 2300 bp paired end mode.

Sequencing reads were demultiplexed in MiSeq reporter v. 2.3 (Illumina). Further data processing was performed in CLC Genomics workbench v. 21.0.2 (Qiagen). Sequence reads were adapter trimmed, filtered, and operational taxonomic unit (OTU)-classified using the Data QC and OTU clustering workflow of the Microbial Genomics Module with default parameters, except 10% of the median coverage was set as cut-off for the filtering of samples based on their number of reads. The Greengenes v.13_5 97% database (available from http://greengenes.secondgenome.com (accessed on 18 January 2017)) was used for classification of 16S datasets, while the UNITE v.7.2 (2017-06-28) 97% with singletons database (available from https://unite.ut.ee/repository.php (accessed on 23 August 2017)) was used for classification of the ITS datasets. The resulting OTU tables were filtered to remove all unknown reads. Reads classified as “chloroplast” at the class level in the 16S data analysis were omitted, since they represent plant/algae sequences and not bacteria.

### 2.4. Isolation and Identification of Cultivable Microbes from Decaying Wood

Wood pieces for microbial isolation studies were harvested and processed following the protocol previously described by Velasco-Rodríguez et al. in 2021 [39]. Supplementary Table S1 lists all the collected samples. In brief, (i) wood pieces were divided into three sections—bark, cambium, and inner part (pith, heartwood and sapwood)]; (ii) the sampling area (bark, cambium) was scraped, whereas the inner section was drilled to obtain sawdust; (iii) serial dilutions in NaCl (0.9%) were produced and plated onto selected media (see below); (iv) plates were incubated in darkness at 25 °C for 3–7 days; (v) the morphologically different colonies were individually transferred to appropriate media, such as: Tryptic Soy Agar (TSA (tryptone soy broth)(Labbox Labware, S.L., Premià de Dalt, Spain): 30 g/L, agar 15 g/L) or Nutrient Agar (NA (nutrient agar powder, PanReac AppliChem, Darmstadt, Germany): 8 g/L, agar 15 g/L)) for the bacterial candidates, and Potato Dextrose Agar (PDA: potato dextrose agar powder (Pronadisa, Torrejón de Ardoz, Spain) 39 g/L, agar 15 g/L) for fungi.

The selected media used in step iii of the isolation process for bacteria were: (i) R2A (yeast extract 0.5 g/L, protease peptone (Fluka, Dresden, Germany) 0.5 g/L, casamino acids (BD) 0.5 g/L, glucose (LAISA) 0.5 g/L, soluble starch 0.5 g/L, pyruvate acid (Sigma) 0.3 g/L, K_2_HPO_4_ (Scharlau, Barcelona, Spain) 0.3 g/L, MgSO_4_·7H_2_O 0.05 g/L, agar 15 g/L), and (ii) Nutrient Agar (NA). Whereas the fungal media were: (i) Potato Dextrose Agar (PDA), (ii) Mold Inhibitory Agar [MIA: dextrose (Pronadisa) 5 g/L, yeast extract (Labkem) 5 g/L, casein peptone (BD) 3 g/L, meat peptone (Scharlau) 2 g/L, sodium phosphate (MERK) 2 g/L, soluble starch (Scharlau) 2 g/L, dextrin (Sigma) 1 g/L, MgSO_4_·7H_2_O (MERK) 0.8 g/L, manganese sulfate (MERK) 0.16 g/L, ferrous chloride (Panreac) 0.04 g/L, sodium chloride (Sigma) 0.04 g/L, agar (ROKO) 15 g/L) and (iii) Malt Extract Agar (MEA: malt extract powder (CultiMed) 19 g/L, agar 15 g/L)).

DNA was isolated using the Wizard™ Genomic DNA Purification Kits (Promega, Stockholm, Sweden) following the supplier instructions. PCR amplification and subsequent sequencing of 1000–1500 and 400–600 nucleotides were carried out for the 16S rRNA and the ITS regions for bacteria and fungi, respectively (PCR primers: Table 2). Consensus sequences of each strain were obtained by using the Seqman tool (DNASTAR Lasergene software package), and identification was done through the GenBank and EMBL databases by BLAST (https://blast.ncbi.nlm.nih.gov (accessed on 11 July 2018); [40]).

### 2.5. Assessment of Microbial Diversity

Alpha diversity describes the mean species diversity in a site at a local scale, whereas the beta diversity describes the ratio between regional and local species diversity [43].

The Shannon index provides a measure of uncertainty associated with selected variables and can be calculated by: $H=\sum_{i=0}^{n}p_{i}\;log_{2}p_{i}\;$,

The Simpson’s index provides a measure of interspecific encounter [44]: $SI=1-\sum_{i=0}^{n}p_{i}^{2}$, where *n* is the number of features and *p_i_* is the fraction of reads that belong to feature *i.*

The Bray–Curtis distance is a measure that describes the dissimilarity between two populations, and it can be calculated by: $B=\frac{\sum_{i=0}^{n}|X_{i}^{A}-x_{i}^{B}|}{\sum_{i=0}^{n}(x_{i}^{A}+x_{i}^{B})}$, where *n* is the number of OTUs and $x_{i}^{A}$ and $x_{i}^{B}$ are the abundances of OTU *i* in samples A and B, respectively [45]. 

Different measures to can be used to describe the microbial diversity. The alpha and beta diversities were calculated from the OTU classification tables using the Estimate Alpha and Beta Diversities workflow in CLC Genomic Workbench (Qiagen). In the alpha diversity analyses, maximum depths of 10,000 and 5000 were used for rarefaction of the bacteria and fungi, respectively, and the number of points was set to 20. In both cases, data reported were for the maximum levels.

### 2.6. Evaluation of Microbial Antagonism against Wood Decay Fungi (Competitive Bioassays)

Tests were performed to determine the antifungal activity of the isolated strains, as previously described by Velasco-Rodríguez et al. in 2021 [39]. In brief, the isolated Actinobacteria and *Penicillium* strains were seeded on both sides of the reporter species, which were five well-known wood-decaying fungi representing the different types of wood damage: *Aspergillus brasiliensis* and *Penicillium chrysogenum* (moulds with some soft rot ability), *Coniophora puteana* (brown rot), *Trametes versicolor* (white rot), and *Aureobasidium pullulans* (blue stain) [7,46].

To allow the development of both species (antagonist and reporter) on the same plate, two different media were selected: Tryptone Soy Agar (TSA), to perform the bioassays against *A. brasiliensis*, *P. chrysogenum*, and *A. pullulans*; and Yeast Extract Malt Extract (YEME: Yeast Extract 3 g/L, peptone 5 g/L, malt extract 3 g/L, glucose 10 g/L and agar 15 g/L) to perform the bioassays against *C. puteana* and *T. versicolor*.

As growth control (negative control), the different antagonists were spread on plates containing both media (TSA or YEME). The reporter strain cultivated alone (positive control) was used to detect normal growth of the reporter species, without any inhibition.

The bioassay was evaluated by comparing the growth of the reporter species alone versus the growth when a microbial antagonist candidate was present. The intensity of the inhibition was scored from 0 to 2, where “0” represents no inhibitory effect, “1” is a partial effect, and “2” represents a total inhibition of the reporter fungi [39].

### 2.7. Polyketide Synthases and Non-Ribosomal Peptide Synthetases Gene Screening

DNA obtained from the species identification was used for polyketide synthases (PKS) and non-ribosomal peptide synthetases (NRPS) detection. PCR amplification was carried out to detect PKS/NRPS genes in Actinobacteria and *Penicillium* species using different sets of degenerate primers, as updated by Fil et al. [47]. Three different sets of primers were used for Actinobacteria (Table 3): K1F/M6R for targeting PKS I [48], KSF/KSR for targeting PKS II [49], and A3F/A7R for NRPS identification (Table 3) [48].

Five pairs of primers were selected to detect putative PKS variants (four sets of primers) and the NRPS (one set of primers) of the different biosynthetic clusters in *Penicillium* species (Table 4). To enable detection of different types of fungal PKS, four different sets of primers were used. Two of them target KA (ketosynthase-acyltransferase): KAF1F/KAR2R (expected fragment of 700–800 bp) and KAF2F/KAR1R (700 bp). The second group of primers was designed to detect the LC series, which covers two different groups of non-reduced PKS ketosynthase domains (WA-type): LC3F/LC5R, (680 bp) and LC1F/LC2R (720 bp) [50,51,52]. The last primer pair (AUG003F/AUG007R) was designed for NRPS detection by means of a 1100-bp fragment amplification (Table 4) [53].

The total DNA of two well-known secondary metabolite producers were used as positive controls: (i) *Streptomyces* *tsukubaensis*, a tacrolimus producer [54,55] for Actinobacteria, and (ii) *Penicillium chrysogenum AS-P-78*, a penicillin producing fungus, for *Penicillium* [56,57].

### 2.8. Enzymatic Assays: Cellulolytic and Feruloyl Esterase Activity Assays

Each selected fungus was inoculated on Petri dishes containing modified Power medium (PW 2: saccharose 25 g/L, lactose 5 g/L, bacteriological peptone 2.5 g/L, corn steep solids 0.5 g/L, KCl 52 g/L, NaNO_3_ 1 g/L, K_2_HPO_4_ 0.25 g/L, MgSO_4_·7H_2_O 0.275 g/L, NaCl 2 g/L, CuSO_4_·7H_2_O 0.0005 g/L, FeCl_3_·6H_2_O 0.0015 g/L and KH_2_PO_4_ 0.03 g/L [58]) and incubated for 1 week. Then, mycelium was scraped with 3-4 mL of sterile solution of 0.9% NaCl, transferred to 50 mL of liquid medium *Penicillium* Minimal Medium Yeast (PMMY: glucose 40 g/L, NaNO_3_ 3 g/L, yeast extract 2 g/L, NaCl 0.5 g/L, MgSO_4_ 0.5 g/L and FeSO_4_ 0.01 g/L [59]), and grown at 250 rpm and 25 °C. The culture was harvested by centrifugation (10 min, 10,000 g) after 96 h of incubation. The supernatant, obtained by centrifugation, was used directly to test enzymatic activity.

Five different media were used in these analyses: (i) PW 2, (ii) PMMY, (iii) Carboxymethylcellulose 1 [CMC: (CBM: NH_4_NO_3_ 2 g/L, KCI 0.5 g/L, KHPO_4_ 1 g/L, MgSO_4_·7H_2_O 0.5 g/L, FeSO_4_·7H_2_O 0.01 g/L, carboxymethylcellulose 10 g/L, agar 20 g/L and penton; 0.2 g/L [60]], (iv) Cellulose Basal Medium (CBM: NH_4_NO_3_ 2 g/L, KCI 0.5 g/L, KHPO_4_ 1 g/L, MgSO_4_·7H_2_O 0.5 g/L, FeSO_4_·7H_2_O 0.01 g/L, cellulose 15 g/L, agar 20 g/L and penton; 0.2 g/L [61]), and (v) Ethyl Ferulate Medium (EF: a 1% (*w*/*v*) Bacto-agar (BD) sterile solution (50 °C) was supplemented with 1.5 mL of filtered ethyl ferulate (0.05 g/mL of ethanol) and poured into plates [62,63]). CMC 1 and CBM media were used for the cellulase screening. The CMC medium, which contained 1% of carboxymethylcellulose, was used to evaluate the endoglucanases activity. CBM was used for the general screening of cellulases. Wells inside the plates were made using a sterile cork borer and were subsequently filled in with 50-µL of fungal culture supernatants and incubated for 48 h at 25 °C. Enzymatic activity was assessed by staining with Congo-red dye (15-20 min) and subsequent washing with 0.9% NaCl (three times); the Congo-red dye is able to bind the cellulose and CMC structures, so the enzymatic activity was detected visually by the appearance of an unstained halo of degraded cellulose and CMC.

For feruloyl esterase activity screening, the protocol described by García-Calvo et al. was used [63]. In brief, wells were made in EF medium plates supplemented with 0.006% bromocresol purple dye and filled in with 50 µL of fungal culture supernatants. The feruloyl esterase hydrolyses ethyl ferulate into ferulic acid, decreasing the pH, and leading to the formation of a yellow halo in the positive samples.

## 3. Results

### 3.1. Metagenome Analyses of Different Species and Rotting Stages of Wood

In order to gain knowledge about the microbiota present in rotting wood of the Spanish forest, decayed pieces of wood were sampled from different tree or bush species at different rotting stages. The selected target location was a peculiar and well-defined biogeographic region that is recognized as a local mosaic, El Bierzo (*Hoya del Bierzo*) in the province of León (Spain) where Mediterranean and temperate macrobioclimates are both described [33]. Selection of tree species for sampling included hardwood species traditionally used in (i) construction: chestnut (*Castanea sativa*), wild cherry (*Prunus avium*), and walnut (*Juglans regia*); (ii) furniture: oak (*Quercus robur*), common pear (*Pyrus communis*), mulberry (*Morus alba*), olive (*Olea europaea*), and ash (*Fraxinus excelsior*); and (iii) wicker baskets (*Salix viminalis*) [64,65,66,67]. In order to analyse which factor (plant species or degradation level) was most relevant for the microbiota diversity, one sample was collected from different sectors of the same tree (e.g., pear tree—Pe1 and Pe2—see Supplementary Table S1), but with different degrees of decay. Thus, 14 samples of rotting wood (Table S1) were analysed by metabarcode sequencing targeting the V3-V4 of the 16S ribosomal rRNA gene of bacteria and the internal transcribed spacer 2 (ITS2) of fungi (Table 1).

**Figure 1:**
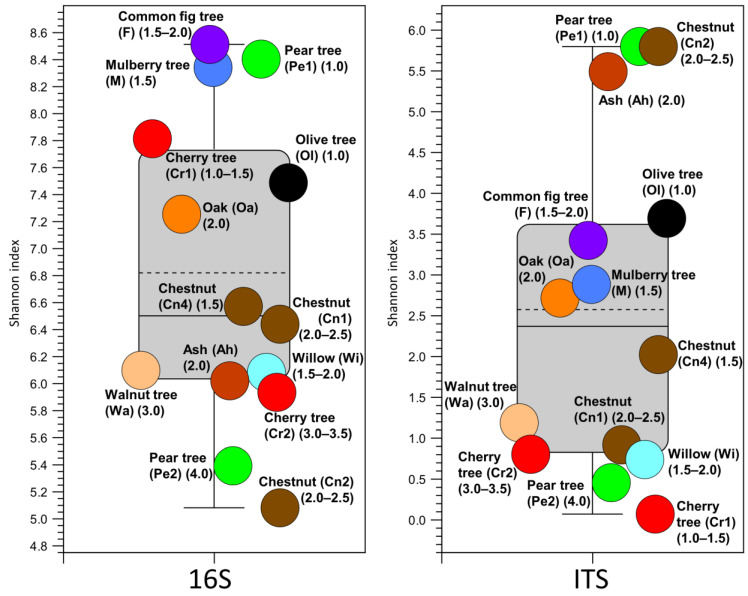
Shannon index at OTU level for bacteria (16S, left) and fungi (ITS, right). Numbers indicate the Swedish National Forest Inventory (NFI) level of wood decay (1, low level of decay; 4, high level of decay), whereas the colours show the tree species. The dashed and full lines show the mean and median values, respectively. The box border percentile is 25, and the whiskers range factor is 1.5.

The estimation of the Shannon and Simpson’s indices, which are well-known measures of biodiversity, showed considerable differences between individual samples in the bacterial level. As a result, no clear pattern was observed related to the tree species. However, the less degraded samples showed a higher Shannon index, suggesting that a more nutritive environment enables the settlement of a larger number of species (Figure 1, Supplementary Figures S1–S3). A similar situation was observed in the fungal samples.

**Figure 2:**
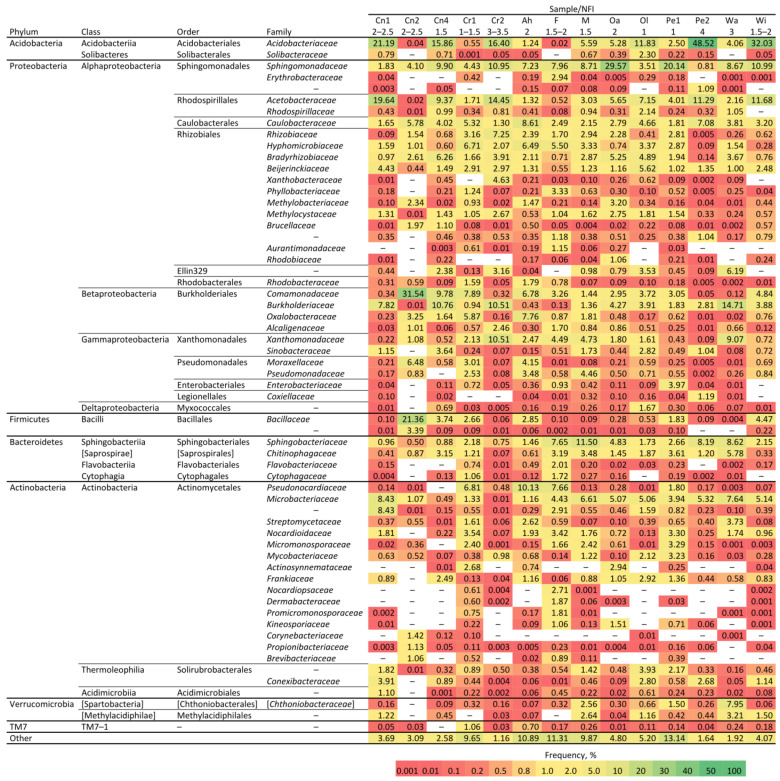
OTU taxonomic analysis at the family level of the bacteria of rotting wood samples. The numbers above the graphic (headed) reflect Swedish National Forest Inventory (NFI) levels (1, low level of decay; 4 high level of decay), whereas the letters indicate the code that was given to the analysed sample (see Figure 1 and Supplementary Table S1). The numbers inside the coloured boxes are frequency percentage of the appearance of a certain family in the sample. The bottom colour scale presents the frequency range of appearance.

The OTU bacterial analysis did not show any conserved distribution of families (Figure 2), but some families were common in most samples, like *Sphingomonadae*, *Comamonadaceae*, *Caulobacteraceae*, *Burkholderiaceae*, *Sphingobacteriaceae*, *Microbacteriaceae*, *Bacillaceae*, and *Rhizobiaceae*. Interestingly, samples with a predominance of the *Acidobacteriaceae* and *Acetobacteriaceae* families showed lower diversity (e.g., the Pe2 sample had 48.52% of *Acidobacteriaceae* and a Shannon index of 5.39, which is the second lowest). The Bray–Curtis distance calculations (Supplementary Figure S4) show that the bacterial microbiota clusters depended more on the degree of rotting than on the host tree species.

**Figure 3:**
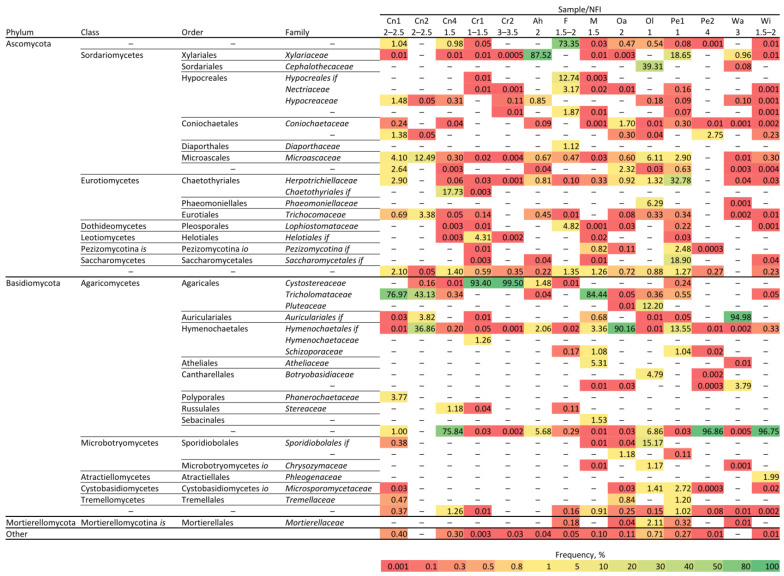
OTU taxonomic analysis at the family level of the fungal fraction of rotting wood samples. The numbers above the graphic (headed) reflect Swedish National Forest Inventory (NFI) levels (1, low level of decay; 4 high level of decay), whereas the letters indicate the code that was given to the analysed sample (see Figure 1 and Supplementary Table S1). Numbers inside coloured boxes are frequency percentage of the appearance of a certain family in the sample. Bottom colour scale presents the range frequency of appearance. Note that the uncertainty at specific taxonomic levels is indicated by *if* (*incertae familiae*), *is* (*incertae sedis*), and *io* (*incerti ordinis*).

The mean and median fungal alpha diversity results (Shannon indices, Figure 1) were lower than the bacterial diversity levels. The level varied considerably between individual samples, but when addressing the OTU distribution (Figure 3), one or a few fungi dominated each sample, and the lower the degree of rotting, the greater the diversity of fungi. The predominant families were *Tricholomataceae* and *Cystostereaceae*. The first one is an extensive fungus family with some species such as *Clitocybula canariensis*, that can cause brown decay [68], whereas *Cystostereaceae* are considered corticioid fungi, which are strongly related to wood decay [69]. For some OTUs, the taxonomic classification is incomplete, and they could only be assigned to the order level (e.g., an unknown family of *Hymenochaetales*) or to the phylum or class level (e.g., an unknown order of Agaricomycetes). These OTUs typically describe microorganisms only detected in metagenome sequencing studies, but which have not been cultivated in the lab; therefore, little or no functional information is known.

### 3.2. Isolation of Cultivable Microorganisms from Decayed Wood

A total of 537 bacterial and fungal species were isolated from the rotten wood samples. Initial isolation was based on morphologic characteristics (reflectivity, colour, texture, size, sporulation, etc). Genetic identification by means of 16S or ITS rRNA regions gave a complete (>98.0% identity) species identification. The sequence alignments identified 343 different fungal strains (fungi and yeast) and 194 different bacterial isolates.

**Figure 4:**
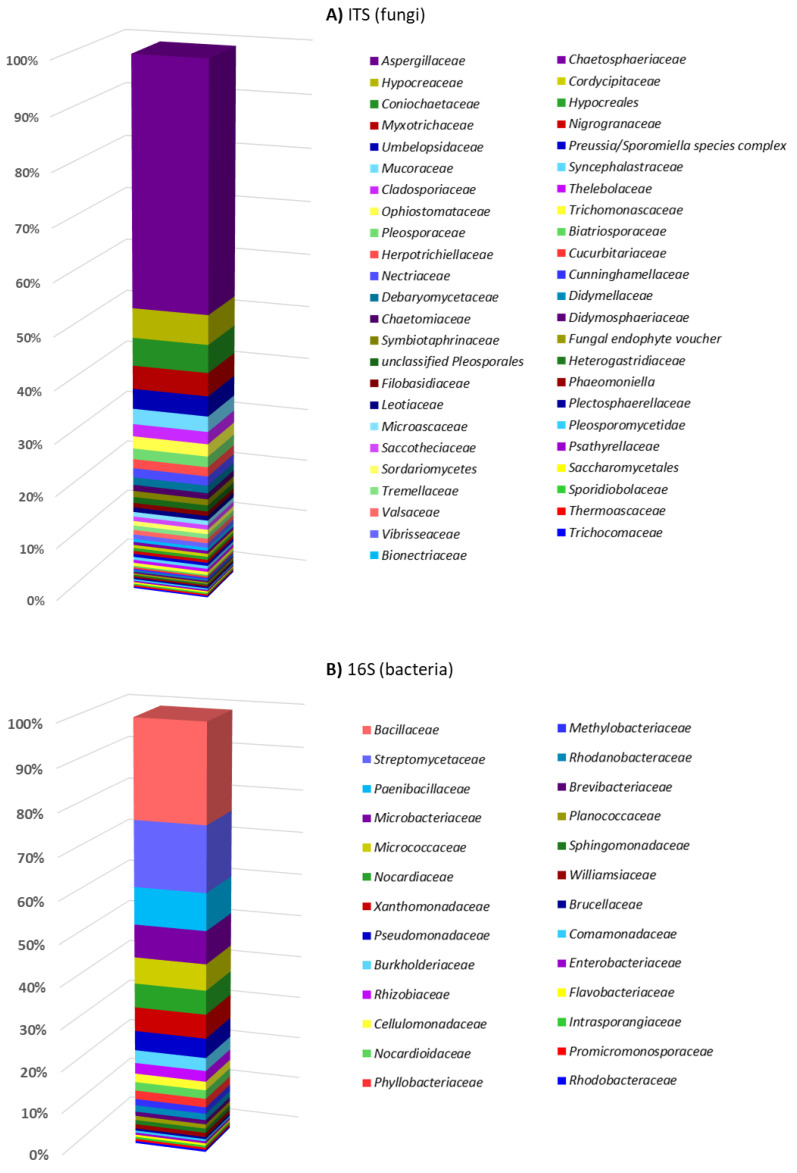
Abundance of microorganisms isolated from wood-decay samples at family level. Each panel presents the family names ordered according to the number of strains isolated from each family: (**A**) fungal families; (**B**) bacterial families.

The 343 fungal isolates were assigned to 3 phyla (Ascomycota, Mucoromycota, Basidiomycota), 29 orders, 47 families (Figure 4A), and 60 genera (one different genus for every 5.7 isolates). Almost half of the fungal isolates belonged to the family *Aspergillaceae*, and within this order, 45.8% belonged to the *Penicillium* genus (156 strains). This is an important genus for the production of bioactive compounds, such as the antibiotic penicillin [70]. The second most identified family was *Hypocreaceae* (5%), which includes *Trichoderma* and *Acremonium* genus. *Trichoderma* species have been applied in the biocontrol of fungal diseases in agriculture and in the bioremediation of organic pollutants [71].

The 194 different bacterial isolates were assigned to 4 phyla (Actinobacteria, Proteobacteria, Firmicutes, Bacteroidetes), 15 orders, 26 families, and 42 genera (one different genus for every 4.6 isolates) (Figure 4B). The predominant family was *Bacillaceae* (23%), which is of interest as a biocontrol and bioremediation agent.

Bacterial species known to produce valuable compounds, such as antibiotics and carotenoids [72], were also abundant (e.g., *Streptomyces* (15%) and *Rhodococcus* (6%)).

### 3.3. Characterization of the Cultivable Isolates: Antimicrobial Potential (NRPS and PKS)

Metabolic fingerprinting analyses showed the presence of different microbial metabolites in the decayed wood, including putative antibiotics (data not shown). The synthesis of these compounds is mediated by complex microbial biosynthetic clusters that are regulated by environmental stimuli (desiccation, lack of nutrients, temperature, etc.). NRPSs are multi-modular enzymes involved in the biosynthesis of many relevant peptide compounds produced by fungi and bacteria [73]. PKS are multifunctional enzymes that catalyse the production of polyketides. These are a large group of bioactive secondary metabolites used as antiparasitic, immunosuppressant, antifungal, antibacterial, or antitumor drugs [47].

*Streptomyces* species are well-known for their ability to produce an impressive array of secondary metabolites [54]. Thus, 76 of 83 actinobacterial isolates were screened for biosynthetic gene clusters. PKSI, PKSII, and NRPKs clusters were detected with degenerate primers using PCR. PKSI was found in 13% (10) of the tested actinobacteria, while PKSII and NRPS, were found in 61% (46) and 70% (53) of the analysed species, respectively. More than half of the tested actinobacteria, 55% (42), contained at least two gene clusters (2 positive hits), and 12% (9) had all the three screened gene clusters (3 positive hits) (Supplementary Table S2).

Among fungi, *Penicillium* was the most frequently isolated genus, regardless of the zone of the wood studied—bark, cambium, or inner part (sapwood and heartwood). As proof of this concept, 32 of the 156 isolated *Penicillium* strains were selected for the screening of antibiotic biosynthetic clusters. Because of the great variability of the PKS and NRPS gene clusters in fungi, five different sets of degenerate primers were used for PCR screening: (i) four pairs for PKSs location (KA series and LC series), and (ii) one pair for NRPSs identification (Table 4). Most of the tested fungi (84%) contained the KA domain of highly reduced PKS. The LC series was detected in 65% of the analysed fungi, but only 3% of the tested fungi contained the NRPS genes (Supplementary Table S3).

### 3.4. Validation of In Vivo Antibiotic Production

Five wood decay fungi were selected as bioassay reporter species for four different wood degradation types: (i) *A. brasiliensis* and *P. chrysogenum* (moulds with some soft rot ability) [46], (ii) *C. puteana* (brown rot), (iii) *T. versicolor* (white rot), and (iv) *A. pullulans* (blue stain). Thus, an easy and fast antifungal activity assay against wood degrader fungi was validated for those Actinobacteria and *Penicillium* species that contained more than one putative antibiotic gene cluster.

**Figure 5:**
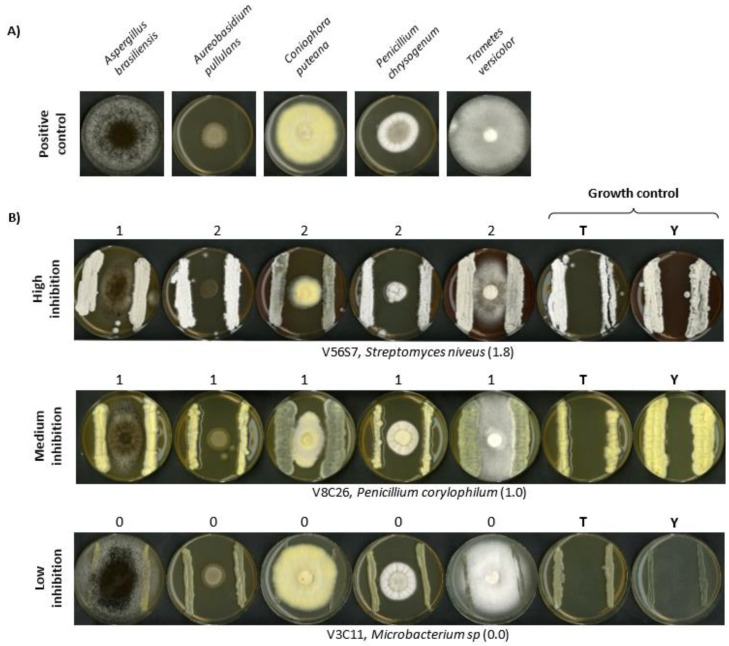
Examples of the evaluation of the antifungal activity in the bioassays. (**A**) Control—reporter species growing on selected medium Trypticase Soy Agar (TSA) or Yeast Extract Malt Extract (YEME) without antagonists (“positive control,” see Materials and Methods): *A. brasiliensis*, *A. pullulans*, *C. puteana*, *P. chrysogenum*, and *T. versicolor*. (**B**) Competitive bioassay with the reporter species in the centre and the antagonistic species at both sides—Actinobacteria (*Streptomyces niveus* or *Microbacterium* sp.) or *Penicillium corylophilum*]. The antagonistic species were plated alone as growth controls (right: growth control) on the selected media TSA (T) and YEME (Y). The bioassay was evaluated by comparing the growth of the reporter species alone versus in the presence of antagonists. The intensity of the inhibition was rated from 0 to 2, where 0 is no inhibitory effect and 2 is total inhibition. The average score of each antagonistic microorganism is shown in parenthesis.

Initially, the antifungal activity of 45 actinobacteria was tested, and 78% of them showed some inhibiting effect (Supplementary Figure S5). A very strong effect was found for 3 (6%), with complete inhibition of the reporter species, whereas 16 species (36%) showed a medium inhibiting capacity, and 26 (58%) had little or no effect on the fungal growth (Figure 5, Supplementary Table S2, and Supplementary Figure S5). Some of the isolated Actinobacteria showed great inhibition capacity, along with the positive identification of an antimicrobial gene cluster. The best example is *Streptomyces niveus* V56S7, which had a score (inhibitory activity against each antagonistic microorganism) of 1.8 and was positive for all the analysed biosynthetic gene clusters (Figure 5 and Supplementary Table S2). *S. niveus* is well-known for its use in the production of novabiocine, a clinically used antibiotic, and marfuraquinocins, which show cytotoxic and antibacterial capacities [74]. 

Nevertheless, this is the first time that high antifungal activity is being reported for this species. Another example is *S. fimicarius* V56S1, which showed high fungal inhibition capacity (1.8) and was positive for PKS-II and NRPS (Supplementary Table S2 and Supplementary Figure S5). Strains of this species have shown antifungal activity due to the production of volatile organic compounds towards the oomycete pathogen *Peronophythora litchi*, which causes downy blight in lychee (*Litchi chinensis* Sonn.) [75]. 

*Penicillium* species that contained three or more positively detected gene fragments were also selected for antifungal activity assay. A total of 16 *Penicillium* isolates were tested. A total of 2 isolates exhibited high inhibition capacity against the fungal reporter strains, *P. expansum* (V8C64) and *Penicillium* sp. (E2S10), 6 isolates (38%) showed a medium effect, while the remaining 5 exhibited a low capacity to repress the growth of wood decaying fungi (Figure 5, Supplementary Table S3, and Supplementary Figure S5).

### 3.5. Enzyme Activities

The ability of enzymes secreted by *Penicillium* strains to degrade different compounds is well known. Thus, the capacity of their secreted enzymes to degrade different molecules present on the lignocellulosic biomass, such as cellulolytic enzymes (exo- and endo-glucanases) or feruloyl esterases, has been described [27,28].

**Figure 6:**
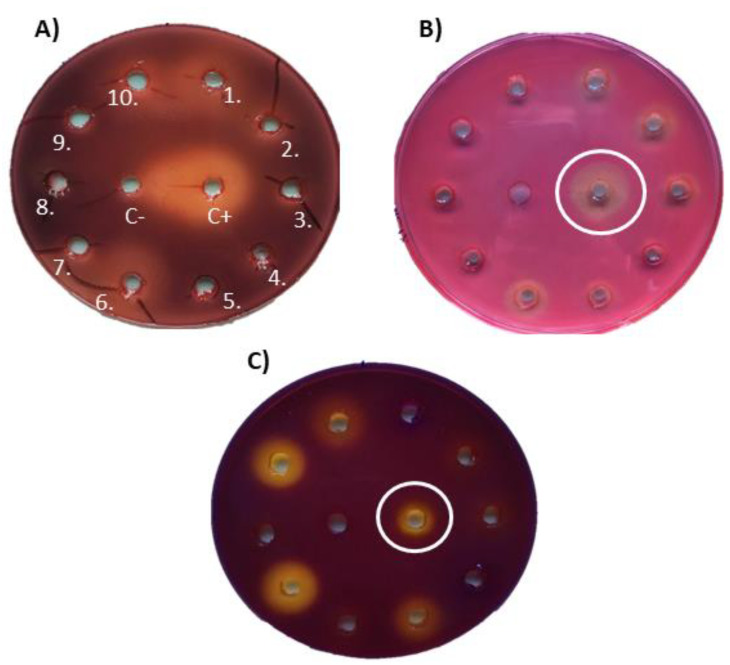
Example of enzymatic activity assays for (**A**) cellulases; (**B**) endoglucanases; (**C**) feruloyl esterases. 1–10: samples; positive control (C+): *Penicillium rubens*; negative control (C-): sterile medium.

Nineteen of the isolated *Penicillium* strains were tested for the production of cellulose degrading enzymes. A total of 16 (84 %) showed endoglucanase activity and 13 (68%) showed exoglucanase activity (Figure 6A,B, Supplementary Table S4). The same *Penicillium* candidates were analysed as putative feruloyl esterase producers, due to the medical and cosmetic applications of ferulic acid, which have increased the interest in these enzymes [63]. Eleven isolates (58%) were positive with respect to this activity (Figure 6C, Supplementary Table S4).

In summary, seven isolates (37%) were positive for all the three tested enzyme activities, suggesting that these *Penicillium* strains may be able to degrade cellulose and hydrolyse ethyl ferulate. A combination of both activities can be used to degrade the plant matrix and extract the trapped ferulic acid.

## 4. Discussion

Rotting wood represents an ecological niche where fungi and bacteria compete for space and nutrients in normal, as well as extreme, environments [76]. The initial attack to degrade the lignocellulosic biomass is presumably performed by fungi, but once they have obtained a small foothold, other microorganisms, and especially bacteria, will join them. Some may attack the wood structure under specific nutritional and environmental conditions, while others live off of the metabolites produced by the primary wood degraders [63,77,78]. The release of useful trapped commodity compounds and bioactive molecules makes decaying wood an interesting source of new enzymatic and metabolic activities, as well as their producers. This is consistent with the microbial need to survive in dense and multispecies environments, where the scarce availability of nutritional resources justifies the development of chemical weapons for ecological warfare [79,80]. The current study analysed the microbial diversity, both by isolation of cultivable fungi and bacteria, and by metagenome analyses in a biogeographic region that is recognized as a local mosaic, El Bierzo (*Hoya del Bierzo*) in the province of León (Spain). This region combines the two main macrobioclimates existing in the whole of the Iberian Peninsula and the Balearic Islands: Mediterranean and temperate. This variability in geology, soil, and bioclimate has given rise to a highly diverse vegetation, which strongly suggests the existence of a rich microbiota [33,81].

In many environmental samples, it is not uncommon that the total bacterial plate count yields less than 1% of the total direct count carried out by microscopic methods [82]. Thus, it is not unexpected that these two approaches (metagenomics and traditional isolation) provided very different, but complementary, pictures of the microbiota in rotting wood, in accordance with similar analyses of microbial degraders of polymers [83], saltern pond environments [84], and the bat gut microbiome [85]. In the current study, bacilli constituted approximately one-third of the bacterial isolates, similar to data presented by Embacher et al. during the analysis of bacteria associated with the fungus *Serpula lacrymans* (the most important indoor wood decaying fungus) [86]. However, in most samples, bacilli OTUs constituted only a small fraction of the bacterial OTUs. This can be explained by the excellent ability of bacilli to grow under the applied selection criteria. Moreover, for the fungal species, the two methods were complementary. Fungi of the order Eurotiales constituted 47% of all isolates, but were scarce in the metabarcoding study, where *Trichocomaceae* and *Cystostereaceae*, which were not represented among the isolates, were the most abundant families.

The bacterial families *Acidobacterioaceae* and *Acetobacteraceae* were among the most frequent OTUs in many of the rotten wood samples and constituted more than 15% of the total bacterial OTUs in 5 of 14 samples. Both families contain many acidophilic species [87], and their prevalence in many samples probably reflects the low pH in rotting wood, often less than pH 5.0 [88,89]. While the bacterial diversity was generally high in the rotten wood samples, many of the samples were strongly dominated by one or a few fungal genera. The genera varied among samples, but within each sample, one or a few genera of fungi frequently dominated. In 10 of 14 samples (71%), one genus constituted more than 70% of the OTUs.

At lower degree of rotting, brown rot fungi were predominant (e.g., *Trapeliaceae*, Ochrolechiacea, Cantarelaceaes). Brown rot causes the most severe loss of wood strength. In very decayed wood, white rot fungi, such as *Schizoporaceae*, *Hydnodontaceae*, or Agaricomycetes, were the dominating fungal species. Usually, white rot fungi can degrade all the three main cell wall components, including the lignin, and the data indicate that lignin degradation occurs late in the rotting process. The analyses strongly suggest that the degradation level (NFI level) is more relevant for the microbiota diversity than for the tree or bush species (e.g., pear tree), which is highly relevant for future isolations. 

Previous works indicated the putative antimicrobial activity of microorganisms isolated from decayed wood [11,12,13,14], which fuelled the present analyses of the microbial inhabitants from this non-traditional resource. This study is focused on the detection of genes encoding PKSs and NRPSs, which are involved in the production of different secondary metabolites, such as antibiotics, and the subsequent validation by means of competitive bioassays. A straightforward method of antibiotics screening from wood was applied to two well-known groups of antibiotic producers, Actinobacteria and the *Penicillium* genus [47]. However, when the antibiotic production was tested in vivo by means of competitive bioassays against wood-degrading fungi, the number of confirmed positives dropped drastically. *Streptomyces* species are prolific sources of valuable products, such as antibiotics or siderophores [90,91], for the pharmaceutical industry. This potential has been confirmed by sequencing their genomes, but these species also have drawbacks: (i) most of the compounds are produced in low titers, (ii) the compounds are not usually produced under standard laboratory conditions, and (iii) several of the biosynthetic gene clusters are cryptic [92]. This may explain the low number of positives observed in the bioassays. Several attempts to activate secondary metabolite gene clusters have been published [93,94]. Moreover, the overexpression of a specific or global regulator, combined with promoter replacement, is also one of the most used strategies to turn on cryptic biosynthetic clusters in fungi [95,96].

New regulations demand innovative ways to protect materials without using environmentally dangerous products. Thus, some new trends in the protection of wooden structures have demanded the use of natural products, like plant extracts or microorganisms, to achieve this goal [97]. The antifungal activity versus wood-rotting activity showed by the Actinobacteria and *Penicillium* strains isolated from decayed wood in this work present them as good candidates for sustainable wood preservatives.

The worldwide enzyme industrial market in 2019 was USD 8.6 billion, where food and beverage applications were up to USD 2 billion in 2016. More than 700 commercial products from over 40 industry sectors (e.g., cleaning, textiles, agriculture, paper, animal health, bioenergy, biodiesel, food) are based on enzymes. Today, half of the industrial enzymes are of fungal origin, due to different factors that make them more attractive than those derived from plants or animals. These factors include the: (i) diversity of catalytic activities, (ii) final yields, (iii) possibility for genetic manipulation, and (iv) affordable culture conditions [98,99]. The conversion of lignocellulosic biomass into fermentable sugars for bioethanol production is one of the fields where fungal enzymes are mandatory. Enzymes used for the degradation of the lignocellulosic biomass have been produced by *Aspergillus* and *Trichoderma* strains, but new investigations are pointing to different strains of *Penicillium* as the new producers of these enzymes [27,28]. Several of the isolated *Penicillium* species demonstrated their capacity to produce enzymes of industrial interest (e.g., 1, 4-β-endoglucanases, cellulases, feruloyl esterases), thus confirming rotten wood as a promising source of enzymatic activities.

## 5. Conclusions

Based on metagenome and classical isolation analyses, decaying wood is shown to be a rich and varied microbial environment, even though the complementary analyses did not correlate properly when cultivable species and metabarcode sequencing results were compared. The diversity of the microbiota seemed conditioned by the degree of decay of the samples (NFI level), which open an interesting field of study.

Intriguingly, the most common isolated microorganisms from rotten wood were well-known antibiotic-producing microorganisms, such as *Penicillium* (45.8% of isolated fungi) or Actinobacteria (40.7% of bacterial isolates). Genetic (PKSs and NRPSs detection) and bioassays analyses confirmed their antifungal activity and their industrial potential. Moreover, the enzymatic potential for the lignocellulose valorisation of several *Penicillium* isolates was probed.

In summary, decaying wood is an unexplored environment that may be a valuable source of industrially relevant bacteria and fungi. Several isolated species, such as *S. niveus* (V56S7), *S. fimicarius* (V56S1), *P. expansum* (V8C64), and *Penicillium* sp. (E2S10) validate the hypothesis of them filling of an interesting microbial niche due to their potential for the secretion of antifungal compounds or enzyme production, and postulate them as suitable cell factories for pharma, food, feed, and cosmetics industries.

## Figures and Tables

**Table 1:** Set of primers used for species identification. PCR primers used for generation of amplicons of metagenomic sequencing libraries of bacteria (16S) or fungi (ITS2). Illumina adapters were added to the original sequence; the priming sequences are highlighted in bold.

Primers	Metabarcode	Ref.	PCR Fragment
16S-1 (Bakt_341F/S-D-Bact-0341-b-S-17)	5′-TCG TCG GCA GCG TCA GAT GTG TAT AAG AGA CAG **CCT ACG GGN GGC WGC AG**-3′	[35,36]	~402–427 bp
16S-2 (Bakt_805R/S-D-Bact-0785-a-A-21)	5′-GTC TCG TGG GCT CGG AGA TGT GTA TAA GAG ACA G**GA CTA CHV GGG TAT CTA ATC C**-3′		
ITS2-1 (ITS4)	5′-TCG TCG GCA GCG TCA GAT GTG TAT AAG AGA CAG **TCC TCC GCT TAT TGA TAT GC**-3′	[37]	~200–300 bp
ITS2-2 (based on fITS7)	5′-GTC TCG TGG GCT CGG AGA TGT GTA TAA GAG ACA G**GT GAA TCA TCG AAT CTT TG**-3′	[38]	

**Table 2:** Set of primers used for species identification PCR primers used for Sanger sequencing to identify cultivable microorganisms (bacteria: 27F/1492R; fungi: ITS1/ITS4).

Primers	Sanger Sequencing	Ref.	PCR Fragment
**27F**	5′-AGA GTT TGA TCC TGG CTC AG-3′	[41]	1000–1500 bp
**1492R**	5′-GGT TAC CTT GTT ACG ACT T-3′	[42]	
**ITS1**	5′-TCC GTA GGT GAA CCT GCG G-3′	[37]	400–600 bp
**ITS4**	5′-TCC TCC GCT TAT TGA TAT GC-3′		

**Table 3:** Sets of degenerate primers for PKSs and NRPS detection in Actinobacteria. Degenerated nucleotides following the International Union of Pure and Applied Chemistry (IUPAC) nucleotide code are indicated.

Primers	Amplicon	Ref.	PCR Fragment
	**Polyketide Synthases (PKSs)**		
K1F	5′-TSA AGT CSA ACA TCG GBC A-3′	[48]	PKSI (1200–1400 bp)
M6R	5′-CGC AGG TTS CSG TAC CAG TA-3′		
KSF	5′-TSG CST GCT TGG AYG CSA TC-3′	[49]	PKSII (600–650 bp)
KSR	5′TGG AAN CCG CCG AAB CCT CT-3′		
	**Non-Ribosomal Peptide Synthetase (NRPS)**		
A3F	5′-GCS TAC SYS ATS TAC ACS TCS G-3′	[48]	NRPS (700–800 bp)
A7R	5′-SAS GTC VCC SGT SCG GTA S-3′		

**Table 4:** Sets of degenerate primers for PKSs and NRPS detection in *Penicillium* sp. Degenerated nucleotides following the International Union of Pure and Applied Chemistry (IUPAC) nucleotide code are indicated.

Primers	Amplicon	Ref.	PCR Fragment
**Polyketide Synthases (PKSs)**			
**KA (ketosynthase** **-** **acyltransferase)**			
**KAF1F**	5′-GAR KSI CAY GGI ACI GGI AC-3′	[50]	PKS (700–800 bp) Highly reduced lovastatin-type PKSs (HR-PKS)
**KAR2R**	5′-CCA YTG IGC ICC YTG ICC IGT RAA-3′		
**KAF2F**	5′-GAR GCI CAY GCI ACI TCI AC-3′	[50]	PKS (700 bp) Methylsalicylic acid synthase type (MSAS-type)
**KAR1R**	5′-CCA YTG IGC ICC RTG ICC IGA RAA-3′		
**PKS ketosynthase domains**			
**LC3F**	5′-GCI GAR CAR ATG GAY CCI CA-3′	[52]	PKS (680 bp)
**LC5R**	5′-GTI GAI GTI GCR TGI GCY TC-3′		
**LC1F**	5′-GAY CCI MGI TTY TTY AAY ATG-3′	[52]	PKS (720 bp)
**LC2R**	5′-GTI CCI GTI CCR TGC ATY TC-3′		
	**Non-Ribosomal Peptide Synthetase (NRPS**)		
**AUG003F**	5′-CCG GCA CCA CCG GNA ARC CHA A-3′	[53]	NRPS (1100 bp)
**AUG007R**	5′-GCT GCA TGG CGG TGA TGS WRT SNC CBC C-3′		

## Data Availability

The raw sequencing reads from this study have been deposited in the NCBI Sequence Read Archive under BioProject, accession PRJNA742743.

## Supplementary Materials

The following are available online at https://www.mdpi.com/article/10.3390/microorganisms10061249/s1: Figure S1: Simpson’s index; Figure S2: Scatter plot of the Shannon index; Figure S3: Scatter plot of the Simpson’s Index; Figure S4: Bray–Curtis distance plot; Figure S5: Antifungal activity assays of Actinobacteria and *Penicillium* species; Table S1: Samples collected from Villavieja (León, Spain); Table S2: Screening of PKS/NRPSs genes in Actinobacteria; Table S3: Screening of PKS/NRPSs genes in *Penicillium* sp.; Table S4: Results of cellulolytic and feruloyl esterase activities.
