# Supplementary material for: Characterization of Microbial Diversity in Decayed Wood from a Spanish Forest: An Environmental Source of Industrially Relevant Microorganisms
Source: Microorganisms. 2022 Jun 18;10(6):1249. doi: 10.3390/microorganisms10061249 (PMC9227542; doi:10.3390/microorganisms10061249)
Supplement: Supplementary file 1 [file microorganisms-10-01249-s001.zip › Supplementary Material Figures.pdf]

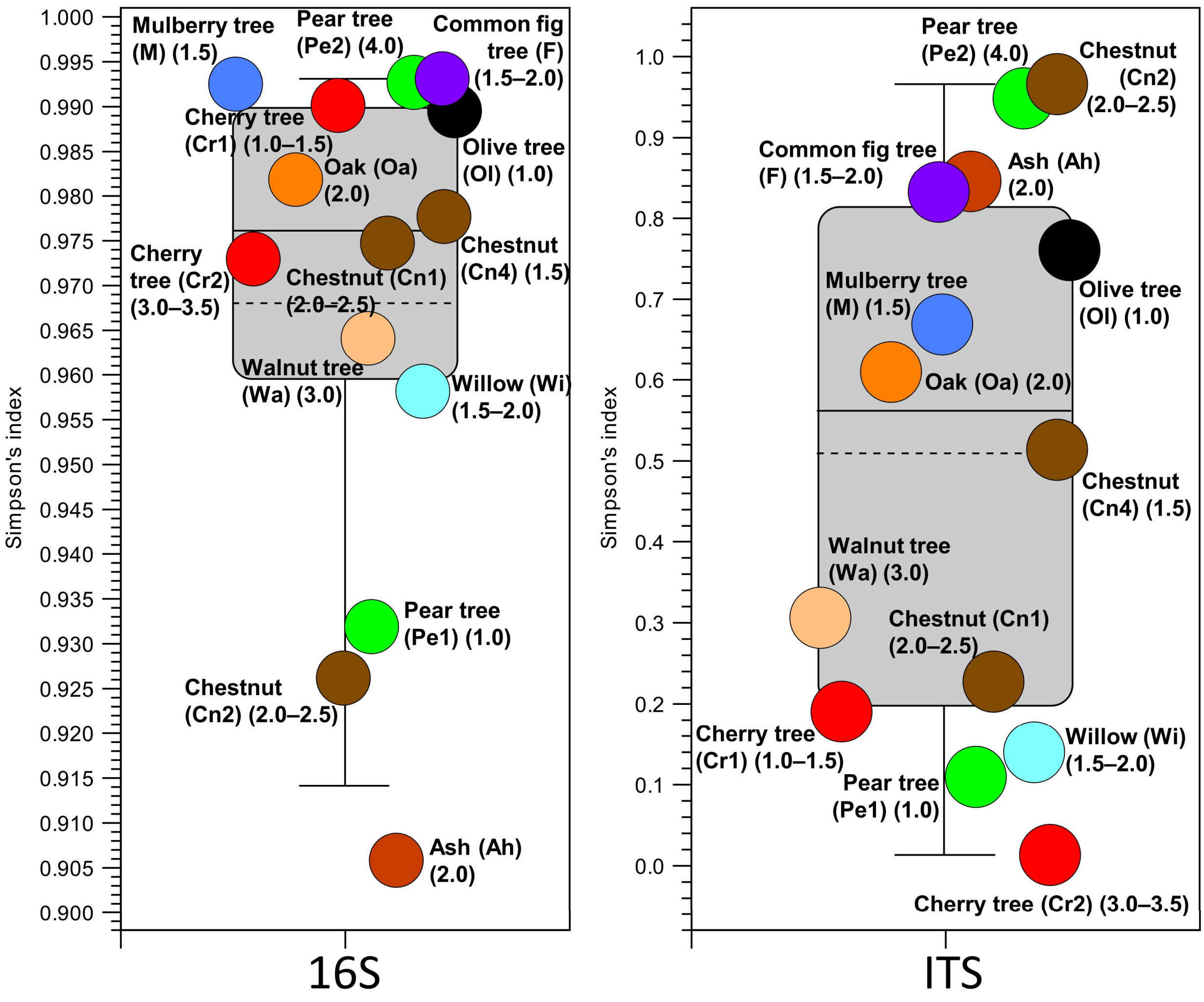

Supplementary Figure S1: Alpha diversity, Simpson's index at the OTU level. Numbers reflect NFI levels (1: low level of degradation; 4: high level of degradation)), whereas the colours show the tree species. The dashed and full lines show the mean and median values, respectively. The samples codes, on brackets, are summarizes on the Supplementary Table S1.

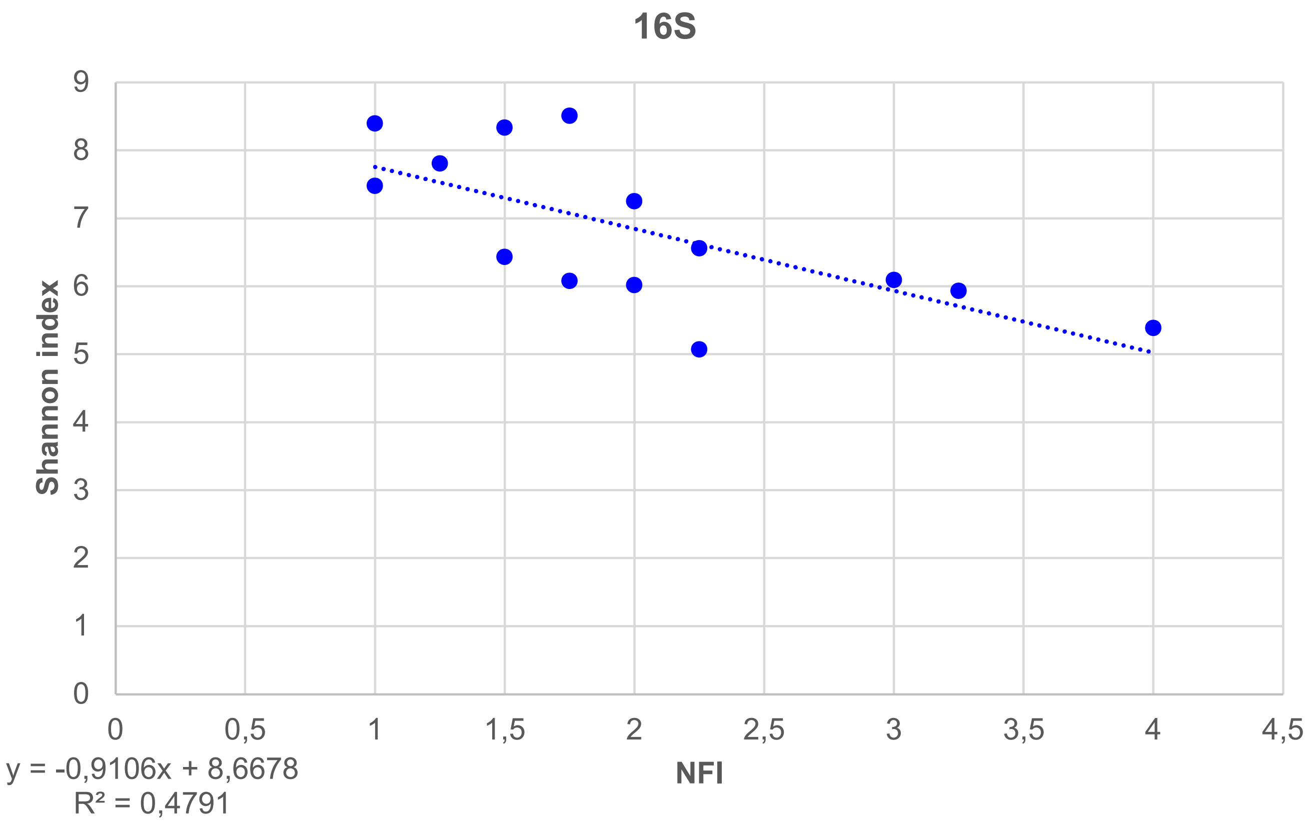

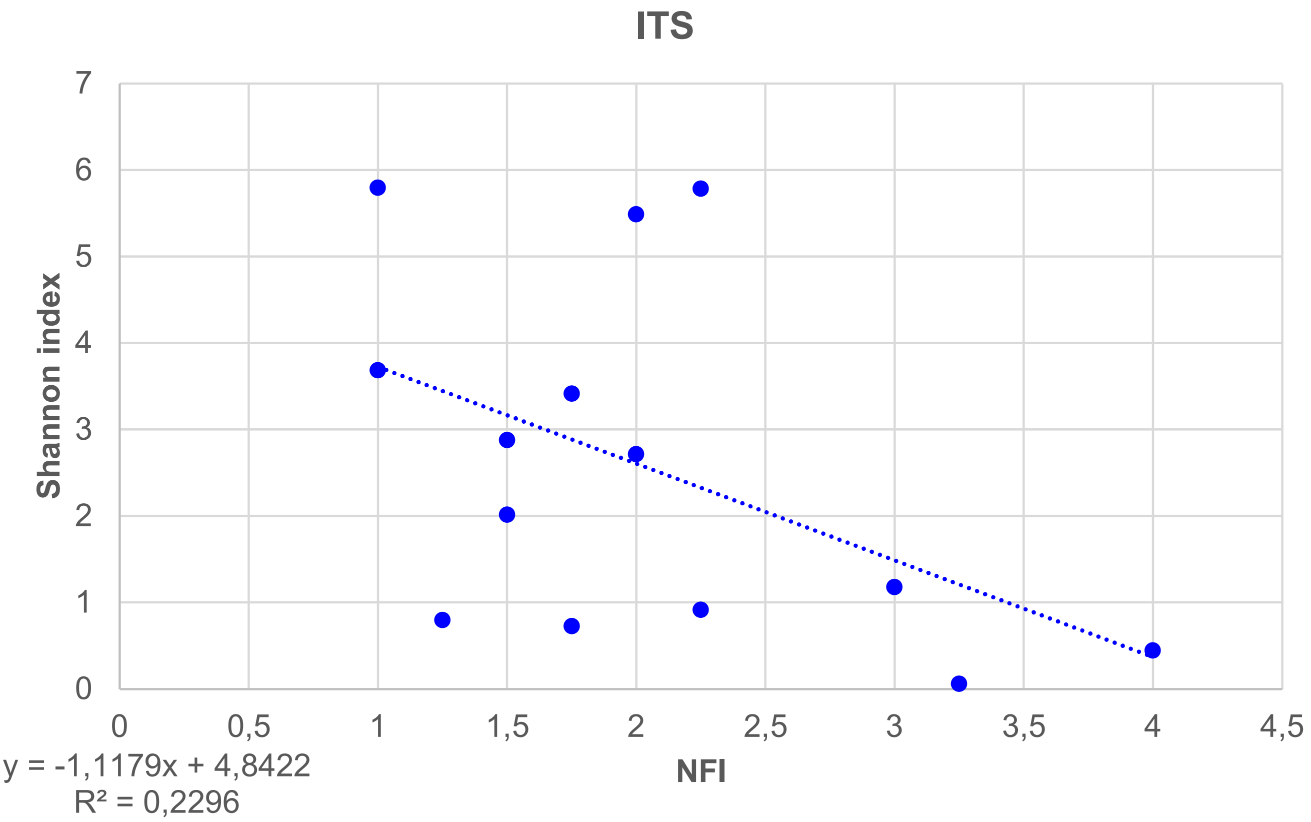

Supplementary Figure S2: Scatter plot of Shannon Index. Numbers reflect NFI levels (1: low decay; 4: high decay). Top panel shows data based on the bacterial fraction while the bottom panel shows the fungal fraction

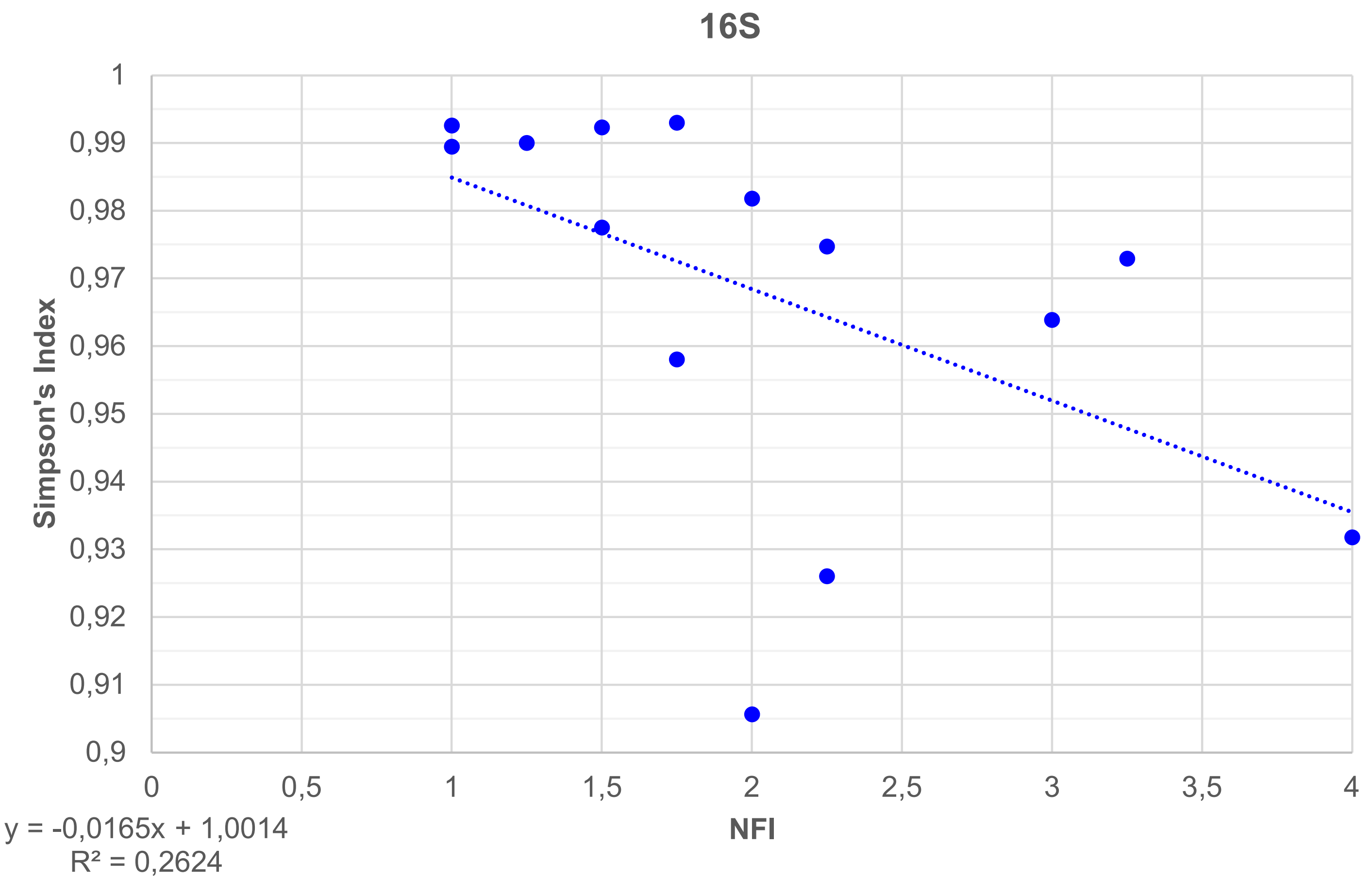

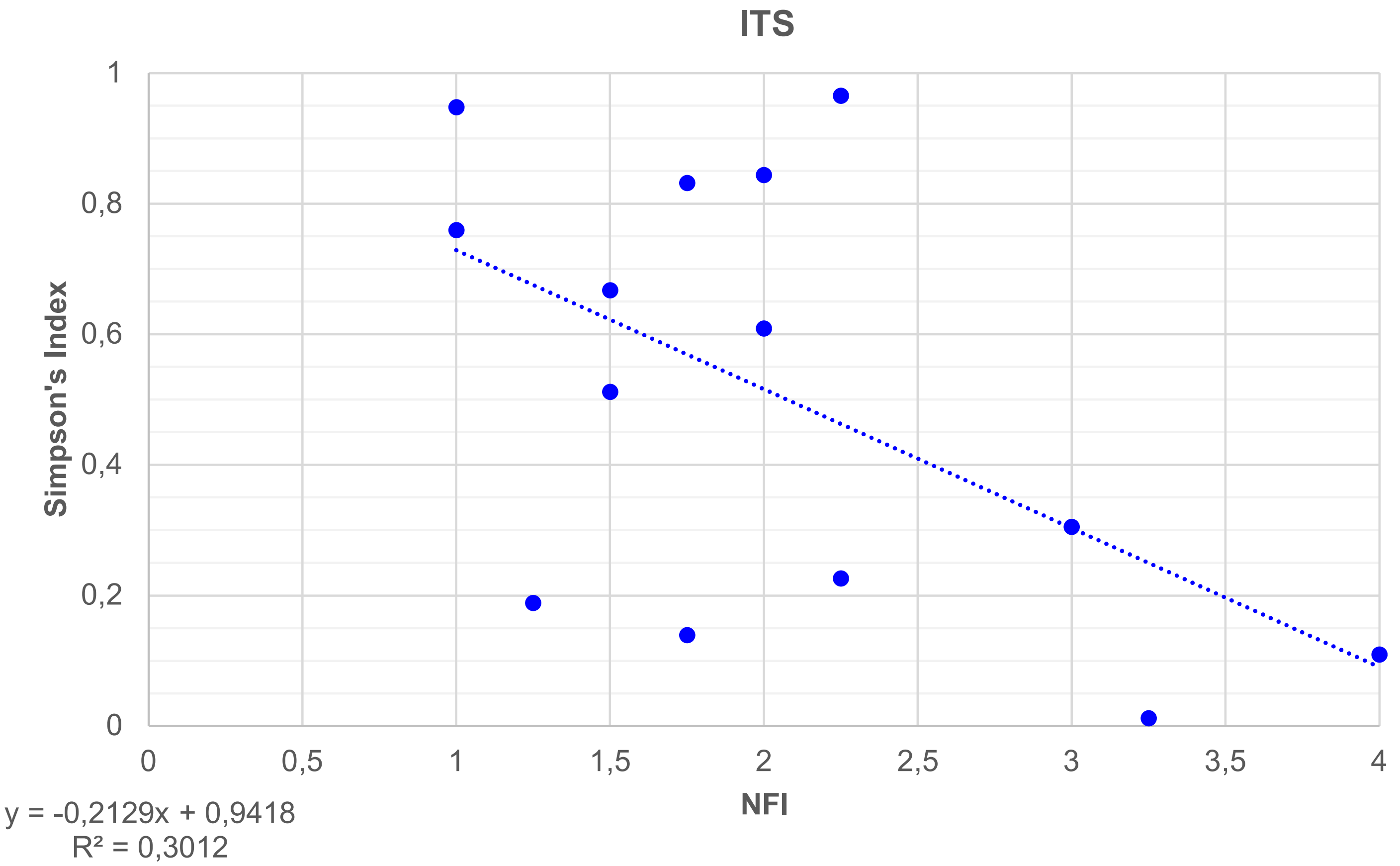

Supplementary Figure S3: Scatter plot of Simpson's Index. Numbers reflect NFI levels (1: low decay; 4: high decay). Top panel shows data based on the bacterial fraction while the bottom panel shows the fungal fraction

16S (Bacteria)

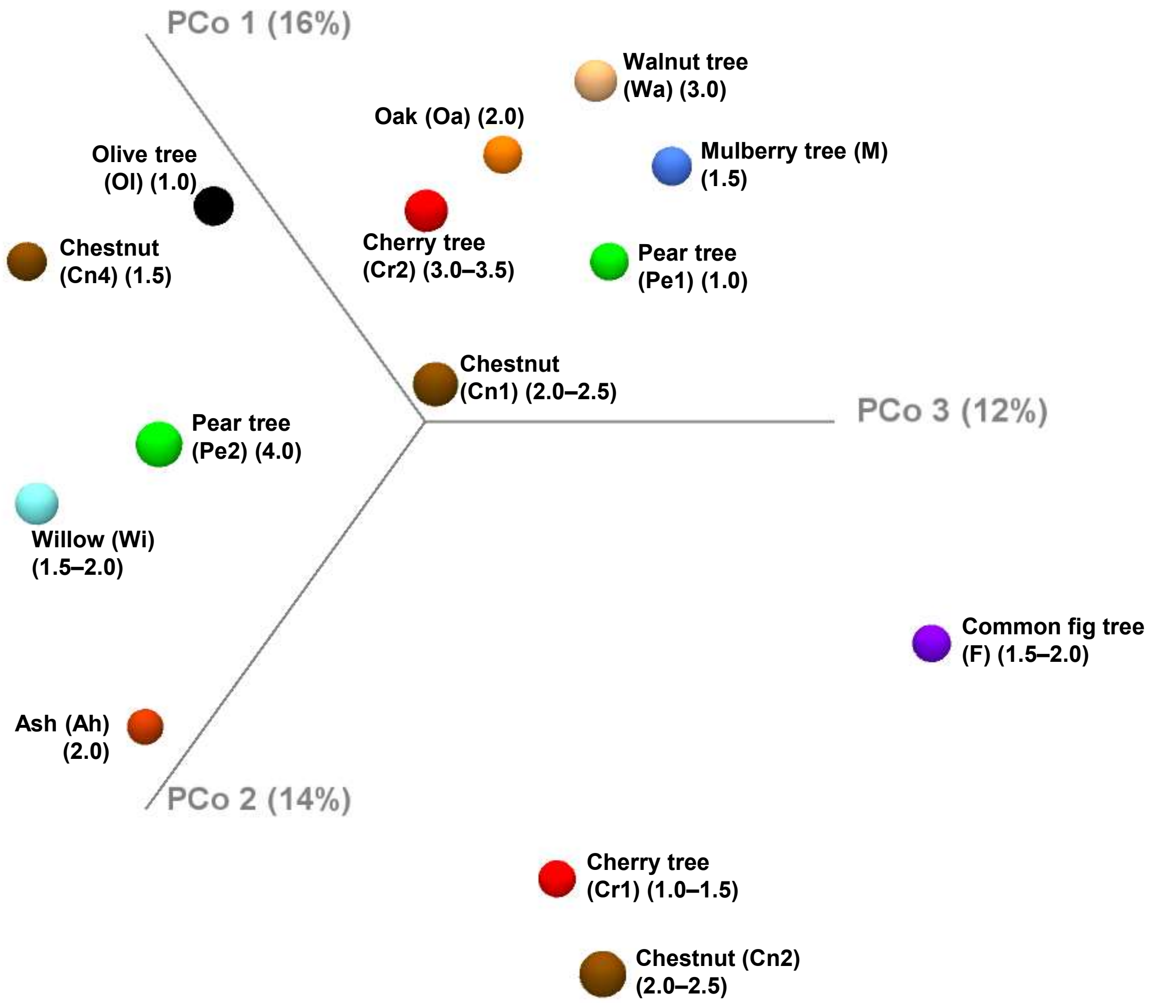

ITS (Fungi)

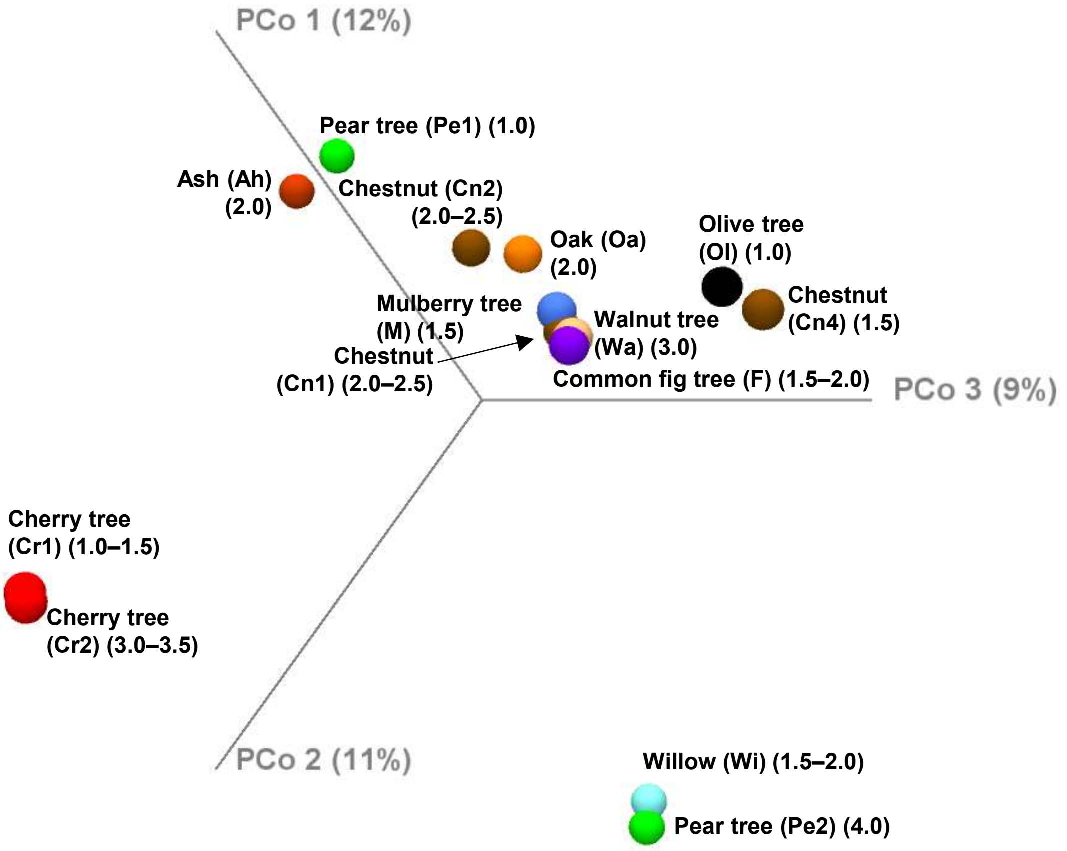

Supplementary Figure S4: Bray-Curtis distance plot. Samples have been coloured like in Figure 1, reflecting the tree origin of each sample. Numbers reflect NFI levels (1: low decay; 4: high decay). Top panel shows data based on the bacterial fraction while the bottom panel shows the fungal fraction.

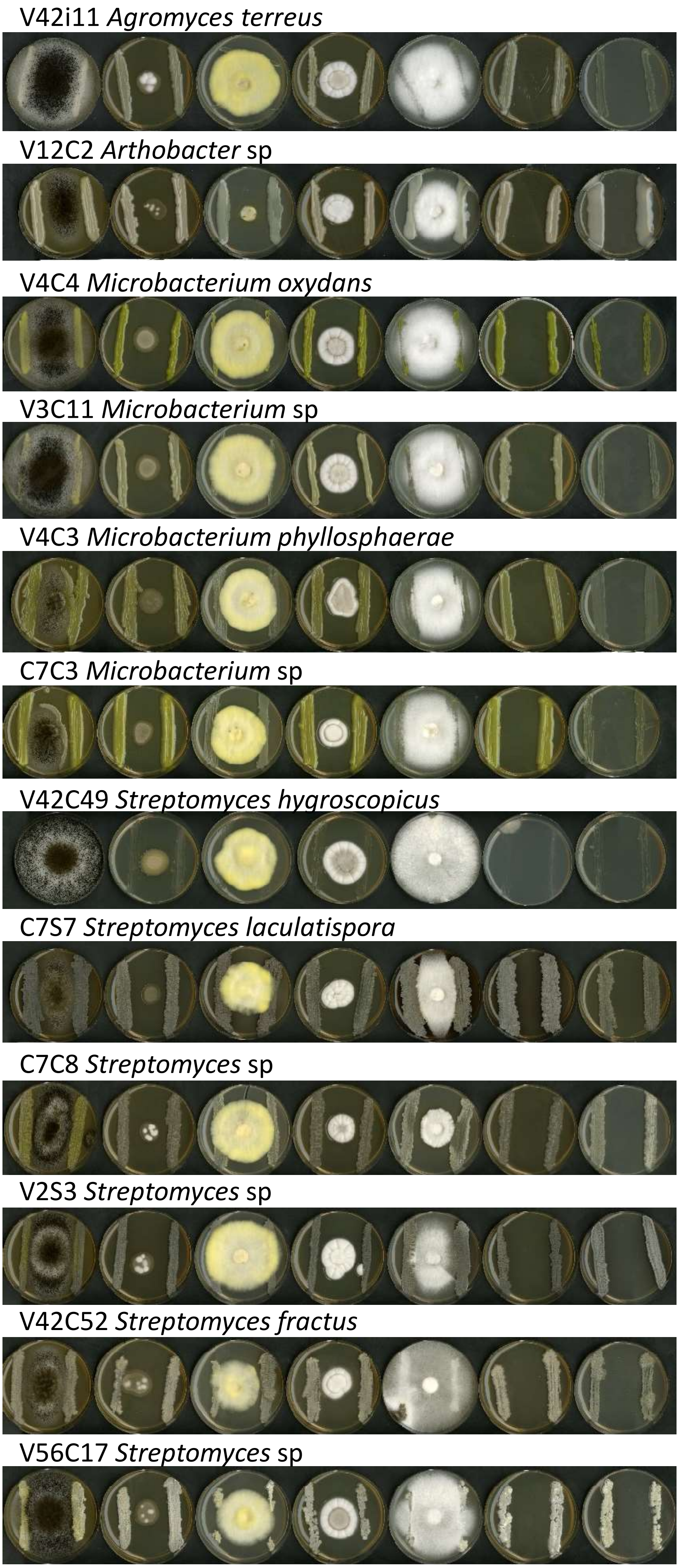

Supplementary Figure S5: Actinobacteria and *Penicillium* strains antifungal activity assays with the reporter species in the centre (in order of appearance, from right to left; *Aspergillus brasiliensis*, *Aureobasidium pullulans, Coniophora puteana*, *Penicillium chrysogenum* and *Trametes versicolor*) and the antagonistic species at both sides. The antagonistic species were plated alone as growth controls (on the right) on the selected media TSA and YEME.

V5C2 *Streptomyces* sp

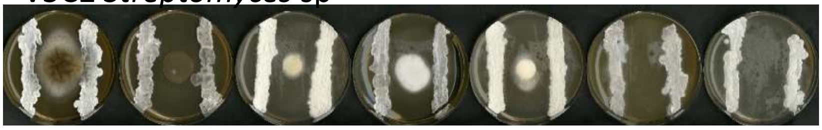

V5i1 *Streptomyces kanamyceticus*

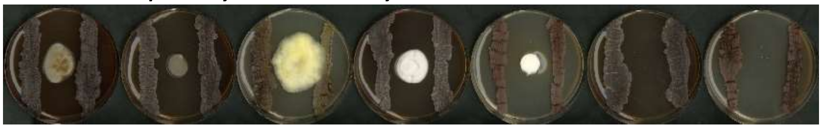

V5i2 *Streptomyces* sp

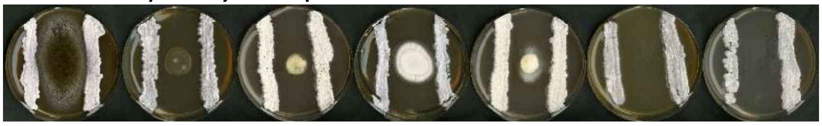

V5S6 *Streptomyces lavendulae*

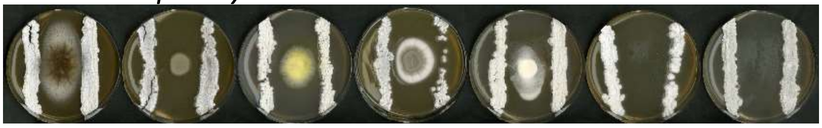

V5S7 *Streptomyces* sp

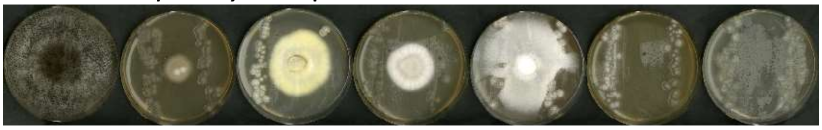

V56S7 *Streptomyces niveus*

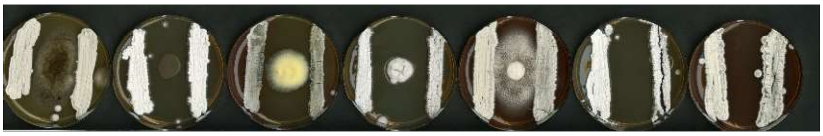

V42C53 *Streptomyces lividans*

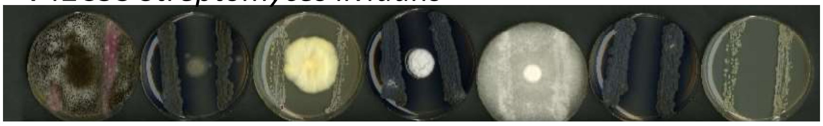

V56S1 *Streptomyces fimicarius*

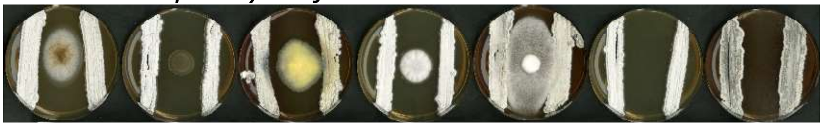

C7i7 *Streptomyces* sp

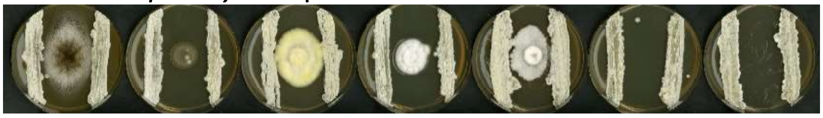

V17C6 *Streptomyces* sp

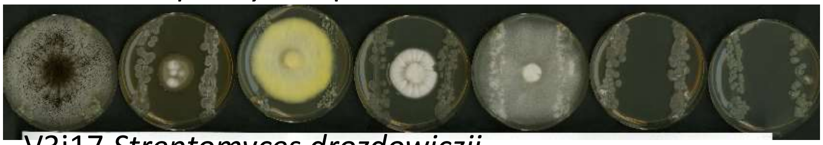

V3i17 *Streptomyces drozdowiczii*

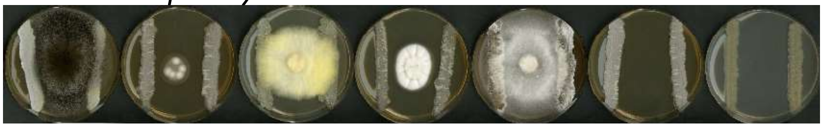

V36C4 *Streptomyces flavogriseus*

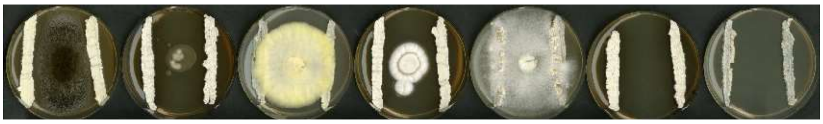

Supplementary Figure S5: Cont.

V42C66 *Penicillium aenum*

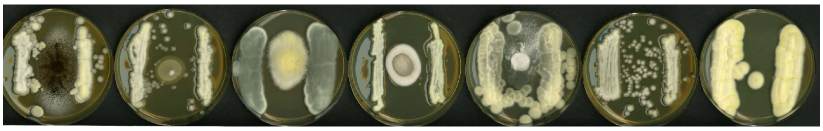

V42C63 *Penicillium charlesii*

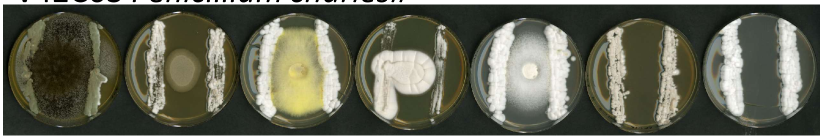

V8C26 *Penicillium corylophilum*

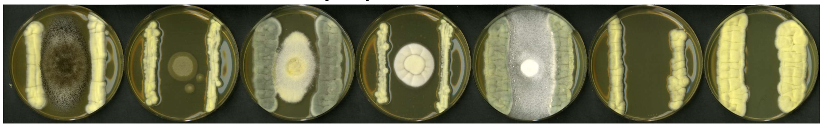

V8C31 *Penicillium decaturense*

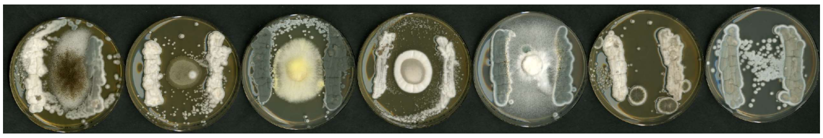

V8C64 *Penicillium expansum*

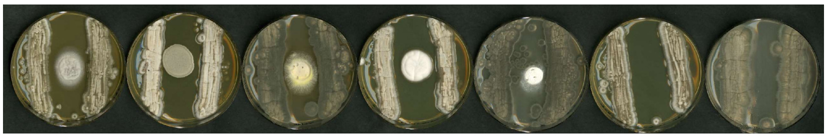

V8C126 *Penicillium toxicarium*

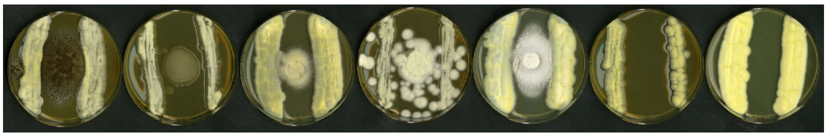

V4C32 *Penicillium* sp

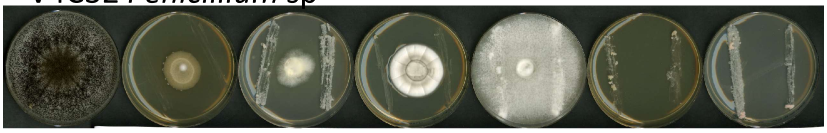

E2C22 *Penicillium citrosulfuratum*

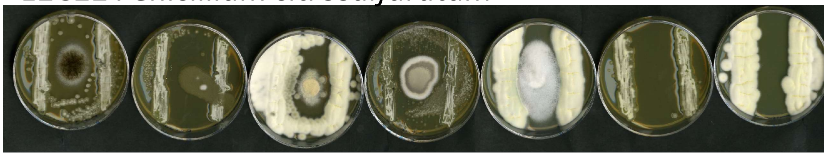

V12S13 *Penicillium brevicompactum*

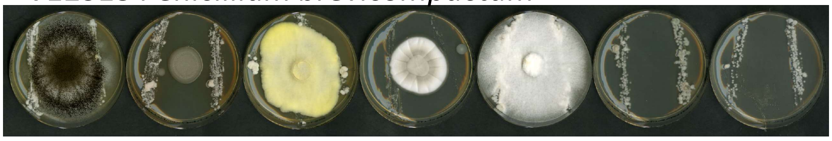

V8i77 *Penicillium murcianum*

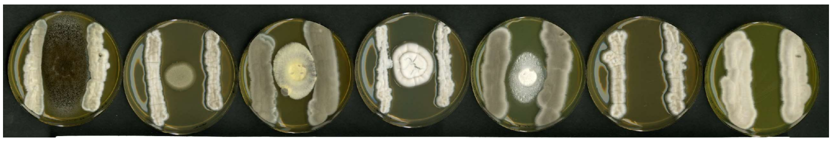

V4S2 *Penicillium* sp

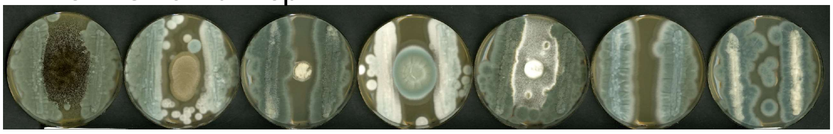

Supplementary Figure S5: Cont.
