# Supplementary material for: Characterization of Microbial Diversity in Decayed Wood from a Spanish Forest: An Environmental Source of Industrially Relevant Microorganisms
Source: Microorganisms. 2022 Jun 18;10(6):1249. doi: 10.3390/microorganisms10061249 (PMC9227542; doi:10.3390/microorganisms10061249)
Supplement: Supplementary file 1 [file microorganisms-10-01249-s001.zip › Supplementary Material Tables.pdf]

SUPPLEMENTARY MATERIAL

Supplementary Table S1: Samples collected from Villavieja (León, Spain: 42.485015, -6.684764, 765 MASL) in June 2017 used for sequencing and microbial isolation. Tree or bush identifier, common name, family and genus of tree or bush, degree of degradation (based on: Swedish National Forest Inventory, NFI), and number of species isolated from each sample and percentage over the whole isolates are indicated. Note that one tree generates more than one analyzed sample (e.g.: Pe has two samples).

Sample	Common name	Family, genus	NFI	No. of isolates (%)
**Pe (Pe2)**	Common pear	Rosaceae, *Pyrus*	4.0	14 (1.9)
**Cn1**	Spanish chestnut	Fagaceae, *Castanea*	2.0-2.5	57 (7.9)
**Ol**	European olive	Oleaceae, *Olea*	1.0	24 (3.3)
**Oa**	European oak	Fagaceae, *Quercus*	2.0	36 (5.0)
**Wi**	Basket willow	Saliaceae, *Salix*	1.5-2.0	55 (7.6)
**Pe (Pe1)**	Common pear	Rosaceae, *Pyrus*	1.0	29 (4.0)
**Ah**	European ash	Oleaceae, *Fraxinus*	2.0	15 (2.1)
**Wa**	English walnut	Juglandaceae, *Juglans*	3.0	109 (15.1)
**M**	Common mulberry	Moraceae, *Morus*	1.5	40 (5.6)
**Cn2**	Spanish chestnut	Fagaceae, *Castanea*	2.0-2.5	12 (1.7)
**Cn4**	Spanish chestnut	Fagaceae, *Castanea*	1.5	43 (6.0)
**F**	Common fig	Moraceae, *Ficus*	1.5-2.0	35 (4.9)
**Cr1**	Wild cherry	Rosaceae, *Prunus*	1.0-1.5	39 (5.4)
**Cr2**	Wild cherry	Rosaceae, *Prunus*	3.0-3.5	31 (4.3)

Supplementary Table S2: Screening of PKS/NRPSs genes in Actinobacteria and results of antifungal activity bioassay. The presence of absence of the biosynthetic genes detected by PCR is coded as, "**+**" PCR amplification and "**-**" no PCR amplification. The average score is the media of the results of each antagonistic specie *versus* the reporter species (*Aspergillus brasiliensis*, *Aureobasidium pullulans, Coniophora puteana*, *Penicillium chrysogenum* and *Trametes versicolor*). The intensity of the inhibition was rated from 0 to 2, where 0 is no inhibitory effect and 2 is total inhibition.

Species	Code	PKS-I K1F/M6R	PKS –II KSF/KSR	NRPS A3R/A7R	Average score
***Agromyces* sp.**	V3C5	-	-	-	-
Agromyces terreus	V42i11	-	+	+	0.0
Agromyces terreus	V42i12	-	-	+	-
Arthrobacter agilis	V8C9	-	+	+	0.0
Arthrobacter agilis	V12i11	-	+	+	-
Arthrobacter citreus	V12C7	-	+	+	0.4
Arthrobacter rhombi	V2C5	-	-	-	1.2
***Arthrobacter* sp.**	V12C2	-	+	+	1.0
***Arthrobacter* sp.**	V8C6	-	+	-	1.2
Brachybacterium tyrofermentans	V2i6	-	-	-	-
***Brevibacterium* sp.**	C7i1	-	-	-	-
***Brevibacterium* sp.**	V42C46	-	+	-	-
***Cellulomonas* sp.**	C7C14	-	+	+	1.2
Curtobacterium flaccumfaciens	V42C25	-	+	+	0.8
Curtobacterium flaccumfaciens	V2C4	-	+	+	-
Curtobacterium oceanosedimentum	V4C13	-	-	-	-
***Curtobacterium* sp.**	V3C6	-	-	+	-
Janibacter melonis	V42C22	-	+	+	0.8
Kocuria rhizophila	V13S1	-	-	-	-
Leifsonia poae	C7C10	-	+	+	0.0
Leucobacter alluvii	V36S5	-	+	+	1.1
Microbacterium oxydans	V4C4	-	-	-	0.0
Microbacterium oxydans	V8C5	-	+	+	1.3
Microbacterium phyllosphaerae	V4C3	-	-	-	0.4
***Microbacterium* sp.**	V3C11	-	+	+	0.0
***Microbacterium* sp.**	C7C3	-	+	+	0.4
***Microbacterium* sp.**	V12C5	-	+	+	1.1
Micrococcus luteus	V12i9	-	-	-	-
Micrococcus luteus	V5i4	-	-	+	-
Micrococcus luteus	V4i7	-	-	+	-
Micrococcus terreus	V12C4	-	+	-	-
Nocardioides albus	V56S2	-	-	-	-
***Nocardioides* sp.**	C7C9	-	+	+	0.0
Nocardiopsis umidischolae	V3C7	-	-	-	1.4
Oerskovia enterophila	V3i1	-	-	-	-
Oerskovia paurometabola	V3i13	-	-	-	-
Promicromonospora sp.	V56C15	-	-	-	-
Rhodococcus erythropolis	V8i7	-	-	+	0.0
Rhodococcus erythropolis	C7S1	-	-	+	-

Rhodococcus fascians	C7C6	-	+	+	0.8
Rhodococcus globerulus	V2i2	-	-	-	1.2
Rhodococcus globerulus	V2S4	-	+	+	-
Rhodococcus opacus	C7i4	+	-	+	1.0
Rhodococcus **sp.**	V12C3	-	-	+	-
Sediminihabitans luteus	V3C22	-	+	-	-
Streptomyces candidus	V12i3	+	+	+	0.2
Streptomyces drozdowiczii	V3i17	+	+	+	0.2
Streptomyces fimicarius	V56S1	-	+	+	1.8
Streptomyces flavogriseus	V36C4	-	+	+	0.2
Streptomyces flavogriseus	V1i7	+	+	+	-
Streptomyces fractus	V42C52	-	+	+	0.2
Streptomyces griseus	V3i9	-	+	+	0.4
Streptomyces hygroscopicus	V42C49	-	-	-	0.0
Streptomyces kanamyceticus	V5i1	+	+	+	1.4
Streptomyces laculatispora	C7S7	+	+	+	1.0
Streptomyces lavendulae	V5S6	-	-	+	1.0
Streptomyces lavendulae	V56C10	-	-	-	-
Streptomyces niveus	V56S7	+	+	+	1.8
Streptomyces **sp.**	V17C6	-	+	+	0.0
Streptomyces **sp.**	V5S7	-	+	+	0.0
Streptomyces **sp.**	V56C17	-	+	+	0.2
Streptomyces **sp.**	V2S3	+	+	+	0.4
Streptomyces **sp.**	V42C42	-	+	+	0.8
Streptomyces **sp.**	C7C8	+	+	+	1.0
Streptomyces **sp.**	V5i2	-	+	+	1.4
Streptomyces **sp.**	V5C2	-	-	-	1.4
Streptomyces **sp.**	C7i7	-	+	+	1.8
Streptomyces **sp.**	V12S5	-	-	+	-
Streptomyces **sp.**	V42C43	-	+	+	-
Streptomyces **sp.**	V42C48	-	+	+	-
Streptomyces **sp.**	V42C50	-	+	-	-
Streptomyces **sp.**	V42C51	-	+	+	-
Streptomyces thinghirensis	V42C55	+	+	+	0.4
Streptomyces violaceolatus	V42C53	-	+	+	0.8
Williamsia muralis	V12C9	-	-	+	-
Williamsia sp.	C7C13	-	-	+	-

Supplementary Table S3. Screening of PKS/NRPSs genes in *Penicillium* sp. and results of antifungal activity bioassay. The presence of absence of the biosynthetic genes detected by PCR is coded as, "**+**" PCR amplification and "**-**" no PCR amplification. The average score is the media of the results of each antagonistic species *versus* the reporter species (*Aspergillus brasiliensis*, *Aureobasidium pullulans, Coniophora puteana*, *Penicillium chrysogenum* and *Trametes versicolor*). The intensity of the inhibition was rated from 0 to 2, where 0 is no inhibitory effect and 2 is total inhibition.

Species	Code	PKSs				NRPS	Average score
		KAF1F/ KAR2R	KAF2F/ KAR1R	LC3F/ LC5R	LC1F/ LC2R	AUG3/ AUG7	
Penicillium aeneum	V42C66	+	-	-	+	-	1.2
Penicillium melinii	E2C16	+	-	+	+	-	1.4
Penicillium brevicompactum	V12S13	+	-	+	-	-	0.2
Penicillium charlesii	V42C63	+	+	+	+	-	1.4
Penicillium citreosulfuratum	E2C22	+	+	+	+	-	1.0
Penicillium citrinum	V8C33	+	-	+	+	-	1.4
Penicillium corylophilum	V8C26	+	+	+	+	-	1.0
Penicillium crustosum	V12S27	+	-	-	+	-	-
Penicillium decaturense	V8C31	+	-	-	-	-	0.4
Penicillium expansum	V8C64	+	-	-	-	-	1.8
Penicillium fellutanum	V12C22	+	+	-	-	-	-
Penicillium glabrum	C7C19	-	-	-	-	-	-
Penicillium kojigenum	V1C11	+	-	+	+	-	-
Penicillium melinii	E2i14	+	+	-	+	+	-
Penicillium citreonigrum	V42C34	+	-	-	-	-	-
Penicillium murcianum	V8i77	-	-	-	-	-	0.8
Penicillium pancosmium	V8i103	+	-	-	+	-	-
Penicillium raistrickii	V4i29	+	-	-	+	-	-
Penicillium roseopurpureum	C7C18	-	-	-	-	-	-
Penicillium sanguifluum	C7S22	+	+	+	+	-	-
Penicillium spinulosum	C7C21	+	-	-	-	-	-
Penicillium steckii	V8i101	+	+	-	-	-	-
Penicillium toxicarium	V8C126	+	-	+	+	-	1.0
Penicillium ubiquetum	V12C15	-	-	-	-	-	0.0
***Penicillium* sp.**	E2S10	+	-	+	+	-	1.6
***Penicillium* sp.**	V12C13	+	-	+	-	-	1.0
***Penicillium* sp.**	V3i21	-	-	+	-	-	-
***Penicillium* sp.**	V3i25	+	-	+	+	-	-
***Penicillium* sp.**	V4S2	+	-	+	+	-	1.0
Penicillium sp.	V8C58	+	-	-	-	-	-
***Penicillium* sp.**	V8i58	+	-	+	+	-	-
***Penicillium* sp.**	V4C32	+	+	+	+	-	0.4

Supplementary Table S4: Results of cellulolytic and feruloyl esterase activity assays. A clear halo indicating the presence of the enzyme is indicated as “**+**”, and the absence of the halo as “**-**“.

Species	Code	Enzymes		
		1, 4-β-endoglucanases	Cellulases	Feruloyl esterases
Penicillium aeneum	V42C66	+	+	+
Penicillium melinii	E2C16	+	+	+
Penicillium brevicompactum	V12S13	+	+	-
Penicillium charlesii	V42C63	+	+	+
Penicillium citreosulfuratum	E2C22	+	+	+
Penicillium citrinum	V8C33	+	+	+
Penicillium corylophilum	V8C26	+	-	+
Penicillium decaturense	V8C31	+	+	+
Penicillium expansum	V8C64	+	+	-
Penicillium glabrum	C7C19	+	+	-
Penicillium kojigenum	V1C11	+	-	-
Penicillium citreonigrum	V42C34	+	-	-
Penicliium murcianum	V8i77	+	+	-
Penicillium toxicarium	V8C126	+	+	+
***Penicillium* sp.**	E2S10	-	+	+
Penicillium sp.	V12C13	-	-	-
***Penicillium* sp.**	V3i25	+	+	-
***Penicillium* sp.**	V4S2	+	-	+
Penicillium sp.	V4C32	-	-	+
